# DISC: a highly scalable and accurate inference of gene expression and structure for single-cell transcriptomes using semi-supervised deep learning

**DOI:** 10.1186/s13059-020-02083-3

**Published:** 2020-07-10

**Authors:** Yao He, Hao Yuan, Cheng Wu, Zhi Xie

**Affiliations:** State Key Laboratory of Ophthalmology, Zhongshan Ophthalmic Center, Sun Yat-sen University, Guangzhou, China

**Keywords:** Single cell, Transcriptome, Deep learning, Semi-supervised learning, Imputation

## Abstract

Dropouts distort gene expression and misclassify cell types in single-cell transcriptome. Although imputation may improve gene expression and downstream analysis to some degree, it also inevitably introduces false signals. We develop DISC, a novel deep learning network with semi-supervised learning to infer gene structure and expression obscured by dropouts. Compared with seven state-of-the-art imputation approaches on ten real-world datasets, we show that DISC consistently outperforms the other approaches. Its applicability, scalability, and reliability make DISC a promising approach to recover gene expression, enhance gene and cell structures, and improve cell type identification for sparse scRNA-seq data.

## Background

Single-cell RNA sequencing (scRNA-seq) measures transcriptomes at single-cell resolution and is widely used to reveal cell heterogeneity and diversity. One of the major challenges in analyzing scRNA-seq data is excess false zero expressions, named dropouts, which distort gene expression distribution and cause misclassification of cell types [1]. The recent advances in droplet- or combinatorial indexing-based sequencing technologies have dramatically increased the throughput from thousands to over a million of cells in a single experiment, causing more severe dropout problems due to shallow sequencing depth per cell [2–4].

Imputation is a common approach to recover dropout events. Most imputation approaches are model-based that borrow information across cells to predict missing expression values [5–7]. Another related approach is “smoothing” that removes the high-frequency signals, including technical noise and dropouts [8]. More recently, deep learning-based approaches have been developed to overcome the scalability issue by conventional approaches. For example, scVI, scScope, and DCA use deep autoencoder (AE) to learn feature representation to recover dropouts and DeepImpute uses a deep neural network to learn gene patterns [9–12].

Although many imputation approaches have been shown to improve the gene expression structure and downstream analysis to some degree, many challenges exist. (1) Reliability: a recent benchmark study showed that most approaches increased sensitivity of recovery of dropouts by scarifying specificity. Therefore, unexpected false signals or other biases have been introduced by imputation [13]. (2) Applicability: factors such as expression level and distribution, level of noises, and heterogeneity of cells affect the performance of imputation. Approaches based on some specific expression or dropout distribution may only work well on some specific datasets [14, 15]. (3) Scalability: conventional model-based approaches cannot handle large datasets, which however have been common in the field due to increasing throughput of scRNA-seq [16]. Thus, a reliable, applicable, and scalable imputation approach is urgently needed.

While more than 90% of genes in scRNA-seq data are zero-counts and the true and dropout zeros are difficult to distinguish, genes in each cell with detected expression (positive-count genes) are more reliable measurements compared to zeros (zero-count genes). Semi-supervised learning (SSL) approach offers promise when a few labels are available by allowing models to supplement their training with unlabeled data [17]. We hypothesize that SSL can build a reliable imputation algorithm by learning information from both positive- and zero-count genes, which can be treated as labeled and unlabeled data, respectively.

Here, we developed DISC, a novel Deep learning Imputation model with semi-supervised learning (SSL) for Single Cell transcriptomes. DISC integrates an AE and a recurrent neural network (RNN) and uses SSL to train model parameters. SSL enables DISC to learn the structure of genes and cells from sparse data efficiently. We compared DISC to seven state-of-the-art imputation approaches, including four deep learning-based approaches. DISC consistently outperformed the other approaches using comprehensive performance metrics evaluating on ten real-world datasets from four different single-cell platforms. DISC enhanced expression distribution and gene-gene/cell-cell relationship validated by two independent FISH experiments. It accurately recovered dropout events and facilitated downstream analysis such as identification of differentially expressed genes (DEGs) and cell types on all the datasets regardless of different platforms and dropout levels. Furthermore, DISC dealt with ultra-large datasets containing millions of cells and required just a portion of computational cost and RAM that other deep learning-based approaches need. Its reliability, efficiency, and scalability make DISC a promising imputation approach for sparse scRNA-seq data. DISC was implemented in Python and publicly available at https://github.com/xie-lab/DISC.

## Results

### Description of DISC

**Fig. 1:**
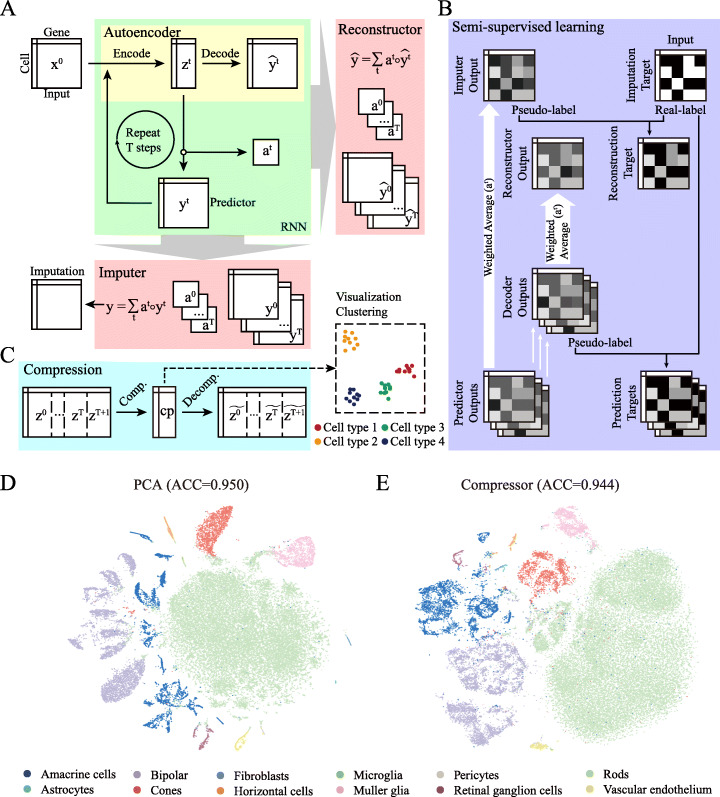
Overview of DISC. **a** DISC contains an autoencoder, a recursive predictor, an imputer to compute an imputation expression profile, and a reconstructor to compute a reconstructed expression profile. **b** DISC is trained in a semi-supervised manner: (1) the imputer learns the expression of positive-count genes, (2) the reconstructor learns both the expression of positive-count genes and the pseudo expression of zero-count genes assigned by the imputer, and (3) the predictors learn both the expression of positive-count genes and the pseudo expression of zero-count genes assigned by the decoder of the same step. **c** Compression module reduces the large latent representations of multiple steps into a much smaller dimension for visualization and clustering. **d** T-distributed Stochastic Neighbor Embedding (T-SNE) visualization and clustering using top 30 PCs generated by PCA transformation from the selected top 2000 highly variable genes (HVGs) of the RETINA dataset (ACC = 0.950). **e** T-SNE visualization and clustering using 50 latent features generated by the compressor of DISC from all 14,871 genes (without HVGs selection) of the RETINA dataset (ACC = 0.944)

DISC has an integrative structure of an AE and an RNN (Fig. 1a). AE is a part of RNN that performs dimension reduction while preserving the manifold of the original data. For each step *t*, the encoder of AE projects the high dimensional cell expression profile (*x*^*t*^) into a low dimensional latent representation (*z*^*t*^). The latent representation is used to predict the cell expression profile through a predictor matrix and to explore the data manifold through the reconstruction of the expression profile by the decoder of AE, obtaining expression profiles from multiple steps either predicted by the predictor (*y*^*t*^) or reconstructed by the decoder of AE ($\hat{y^t}$) (Additional file 1: Fig. S1). Expression profile by the predictor is feed to the next step as the input. At the end, a soft attention framework computes a weighted average of *y*^*t*^ as the imputation result and weighted average of $\hat{y^t}$ as the reconstruction result to support SSL.

Users do not need to specify parameters in the model. Parameters in the layers are automatically learned from data through back-propagation using SSL (Fig. 1b and the “Methods” section). Imputer learns from the positive-count genes using “noise-to-noise” method [18]. Reconstructor learns using SSL from a combination of positive-count genes and zero-count genes assigned a pseudo-count (pseudo-count genes) by imputer to search the best latent representation to reconstruct the expression profile after imputation. Predictor learns using SSL from a combination of positive-count genes and pseudo-count genes assigned by a decoder to search for the best gene expression structure to preserve the manifold learned by AE. This AE-RNN structure enables DISC to learn biological information not only from the small portion of positive-count genes, but also the large portion of zero-count genes.

DISC also provides a solution to compress the latent representation into a lower dimension (50 by default), which retains the most informative information of the expression matrix (Fig. 1c). Ultra-large dataset is beyond the capability of many existing analytical tools. Using the low dimensional representation of the large dataset, clustering and visualization can be performed using existing tools with little comprise in performance. We compared the accuracy of cell-type classification based on the RETINA scRNA-seq data using two dimension reduction methods (Methods), one is the top 2000 highly variable genes transformed to 30 principle components (PCs) by principle component analysis (PCA) and the other is the compressed 50 latent features. The overall classification rates were almost identical (ACCs of 0.950 and 0.944 for the 30 PCs and 50 latent features, respectively), demonstrating the usefulness of the latent representation provided by DISC (Fig. 1d, e).

### DISC is scalable to ultra-large datasets

For large datasets, loading a complete matrix takes a large memory. For example, memory usage is about 100 GB for a matrix with 1,000,000 cells and 10,000 genes. To cope with the large datasets, we designed a novel data reading approach that leverages the ultra-fast chunk reading speed in continuous storage (Methods). As a result, DISC needs a constant initial memory before training, but the memory consumption is stable in datasets with increasing data size.

**Fig. 2:**
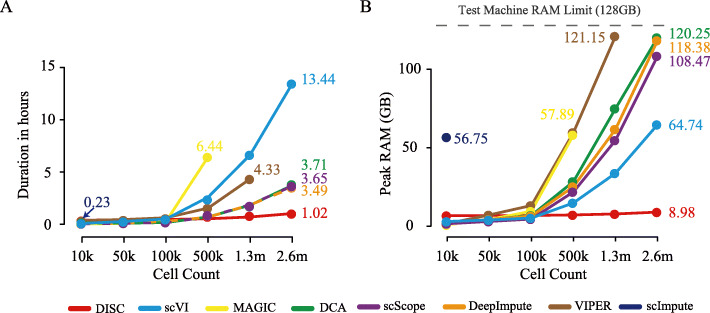
Evaluation of computation usage. **a** Running time and **b** peak RAM usage for datasets with different cell numbers

We compared scalability of DISC with the other imputation approaches on speed and memory usage. We used the 1.3 million (m) mouse brain dataset (BRIAN_1.3 M) as well as datasets with 50 thousand (k), 100 k and 500 k down-sampling cells. We also duplicated 1.3 m cells to 2.6 m cells. All the datasets contained the top 1000 highly variable genes (Methods). As expected, the deep learning-based approaches were much faster and used much less memory (Fig. 2a, b). For the datasets with 10 k, 50 k, and 100 k cells, all the approaches had similar performance except scImpute had much higher memory usage on 10 k dataset and failed on 50 k dataset due to out of memory. MAGIC and VIPER were able to complete the 500 k dataset but took 58 GB memory while five deep learning approaches took less than 25 GB memory. On the 2.6 m dataset, only deep learning approaches could finish the job, where DISC (1.02 h) took less than 1/3 of time took by DeepImpute, DCA, and scScope (3.49, 3.65, and 3.71 h), and 1/13 of scVI (13.44 h). The memory usage of DISC was also considerably less than other approaches. DISC (8.89 GB) took less than 1/7 of memory that scVI needed (65.74 GB) and less than 1/12 that scScope, DeepImpute, and DCA needed (108.47, 118.38, and 120.25 GB).

A previous study showed that the use of less genes inevitably lost information and increased in gene depth to 10,000 genes improved cell-type identification [10]. We tested the imputation performance based on the top 10,000 highly variable genes. DISC was the only approach that can process 1.3 m cells, with 3.2 h and less than 10 GB RAM, and all the approaches encountered “out-of-memory error.” Overall, DISC offers a highly scalable solution for imputation.

### DISC improves gene expression structures validated by FISH

Dropouts severely obscure expression distribution and gene-gene relationship which hinder the downstream analysis [8]. Compared to scRNA-seq, single-cell RNA fluorescence in situ hybridization (FISH) detects a small number of RNA transcripts in single cells and suffers less from dropouts, which is considered a reliable way to validate expression distribution and gene-gene relationship in single-cell levels [6, 12]. To systematically assess DISC’s performance to recover lost gene expression structures by dropouts, we compared imputed expression matrix from scRNA-seq to FISH by three measurements, gene expression distribution measured by root mean square error (RMSE) of Gini coefficient, correlation of gene-gene distributions measured by Fasano and Franceschini’s statistics (FF score), and distance of correlation matrix of gene co-expression measured by correlation matrix distance (CMD). Two independent datasets containing both FISH and scRNA-seq measurements were tested, where the MELANOMA and SSCORTEX datasets have 19 and 33 overlapped genes with FISH, respectively (see the “Methods” section for the description of the datasets).

**Fig. 3:**
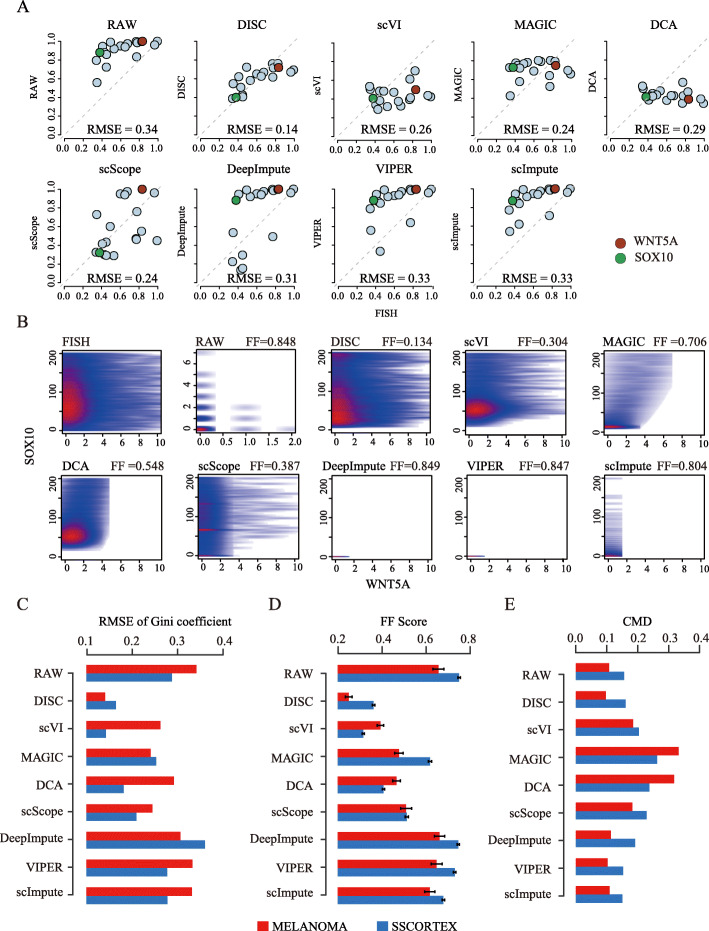
Evaluation of imputation performance by FISH. **a** and **b** were based on the MELANOMA dataset. **a** Scatter plots of Gini coefficients of 19 overlapped genes between FISH and RAW/the imputation, RMSE scores are shown. Two genes with different dropout levels are also shown, WNT5A and SOX10, with dropout levels of 99.85 and 85.18%, respectively. **b** Scatter plot of WNT5A and SOX10 expression levels between FISH and the imputation/RAW. FF scores were calculated across 13,564 cells in FISH and 8498 cells in the other plots. **c** RMSE of Gini coefficients of the RAW/the imputation against FISH in MELANOMA (19 genes) and SSCORTEX (33 genes). **d** FF scores of gene-gene distributions of the RAW/the imputation against FISH in MELANOMA (81 gene pairs) and SSCORTEX (528 gene pairs). **e** CMD of gene co-expression of the RAW/the imputation against FISH in MELANOMA (81 gene pairs) and SSCORTEX (528 gene pairs)

DISC recovered distributions of gene expression across cells on the MELANOMA dataset that resembled the FISH distribution much closer than the raw scRNA-seq data (Additional file 1: Fig. S2, two genes are shown with different dropout levels). For all the 19 genes that had both FISH and scRNA-seq measurements, DISC efficiently improved Gini coefficient (RMSE = 0.14) than the raw scRNA-seq (RMSE = 0.34) and all the other approaches (RMSEs range from 0.24 to 0.33) (Fig. 3a). In addition, DISC recovered the correlation of gene-gene distributions (FF score = 0.134 to FISH) which was lost in the raw data (FF = 0.848) (Fig. 3b, two genes are shown). Indeed, DISC significantly reduced the FF scores for 75 out of the 81 gene pairs (Additional file 1: Fig. S3, *p* < 2.2e−16, one-tailed paired *t* test).

We next compared all the imputation approaches on both the MELANOMA and SSCORTEX datasets. Expression distributions recovered by DISC more closely matched to the FISH compared to the other approaches on MELANOMA and ranked the second on SSCORTEX (Fig. 3c). scVI worked well on one dataset but not well on another. VIPER, scImpute, and DeepImpute did not improve expression distribution compared to the raw data and occasionally even worse (DeepImpute on SSCORTEX). We further evaluated correlations of gene-gene distributions of imputed data to FISH and found that DISC and scVI had the best overall performance on the two datasets (Fig. 3d). VIPER, scImpute, and DeepImpute did not yield improvement compared to the raw data. We also tested how the correlation of gene co-expression in FISH data to the imputed and raw data (Fig. 3e). DISC, scImpute, and VIPER performed well on both datasets while MAGIC and DCA induced substantial false gene co-expression relationship. Altogether, DISC consistently achieved top performance on all measurements of gene expression structure validated by two independent FISH experiments, showing its robust capability to recover gene expression structure obscured by dropouts.

### DISC accurately recovers dropout events

As the true expression of dropouts in scRNA-seq is not possible to obtain, we conducted down-sampling experiments on four datasets (Methods). To test the robustness of imputation performance, we used datasets generated from three different scRNA-seq platforms (Additional file 1: Table S1). Expression matrix before down-sampling (“reference”), after down-sampling (“observed”), and imputation based on the observed were compared.

**Fig. 4:**
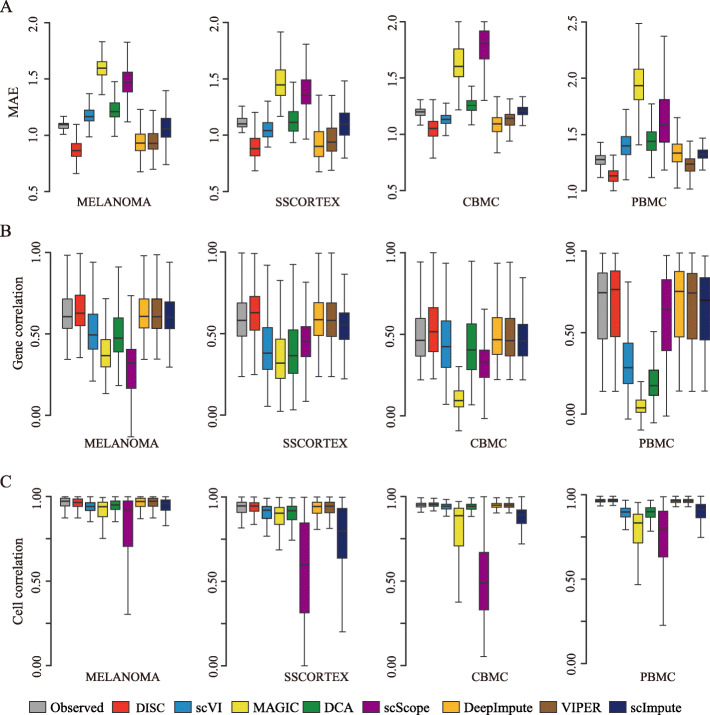
Evaluation of recovery of dropouts in the down-sampling experiments. **a** MAE between the reference and the observed/the imputation. **b** Gene correlation between the reference and the observed/the imputation. **c** Cell correlation between the reference and the observed/the imputation. Box plots show the median (center line), interquartile range (hinges), and 1.5 times the interquartile range (whiskers)

We first measured the accuracy of true gene expression recovery using mean absolute error (MAE) of the imputation to the reference data (Fig. 4a). Notably, DISC achieved the top performance compared to the other approaches on all the datasets. Compared to the observed datasets, DISC significantly recovered gene expression (all the *p* values < 2.2e−16, one-tailed paired *t* test). On the other hand, MAGIC and scScope always performed the worst on all the datasets. We next measured recovery of expression structure using Pearson’s correlation of gene-gene relationship and cell-cell relationship of the imputation to the reference (Fig. 4b, c). For the gene correlation and cell correlation, DISC had the highest correlation coefficients compared to the other seven approaches on all the datasets. It is notable that, for the gene correlation, DISC was the only approach that had improved correlations compared to the observed dataset on all the four datasets while no other approaches had improvement on any dataset, illustrating DISC’s ability to enhance gene-gene relationship. Interestingly, VIPER had almost identical coefficients of cell correlation and gene correlation as the observed data on all the datasets, indicating its strategy to keep the gene structure of the observed data unchanged. DCA and scVI worked well on cell correlation but considerably reduced gene correlation while scImpute performed well on gene correlation but not on cell correlation. MAGIC and scScope significantly reduced both gene correlation and cell correlation compared to the other approaches. In addition, MAGIC and scScope also generated large variations of cell correlation, indicating unstable performance.

We also measured recovery of gene co-expression using CMD of correlation coefficients to assess gene co-expression (Additional file 1: Fig. S4). DISC, scImpute, and VIPER most matched that of the reference, while MAGIC, DCA, DeepImpute, scScope, and scVI generated large false co-expressed relationship for almost all the datasets. This result was consistent with our previous findings using FISH as a validation (Fig. 3e). Collectively, our data showed that DISC consistently and accurately recovered gene expression of dropouts and improved gene structure distorted by dropouts.

### DISC consistently improves cell-type identification

Having demonstrated DISC’s ability to reliably recover dropout events, we next evaluated whether imputation improved cell-type identification. We used three datasets generated from different single-cell platforms, 10X Genomics, Drop-seq, and SPLiT-seq (Methods). We down-sampled the datasets to 30% of the original reads. The average cell library size, reflecting the sequence depth, before and after down-sampling are shown in Additional file 1: Table S1. Percentage of cells correctly assigned (ACC) was used to assess the accuracy of cell-type classification using the marker genes shown in Additional file 1: Table S2 - S4.

**Fig. 5:**
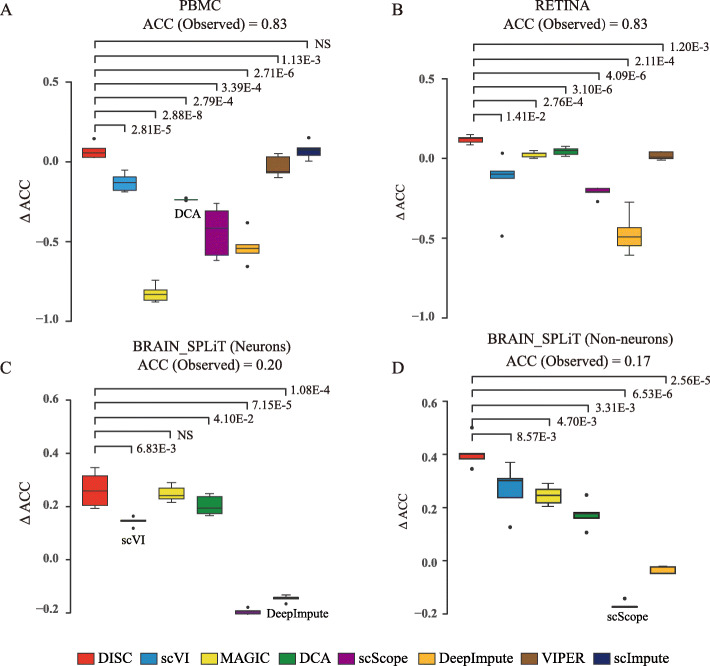
Evaluation of cell type identification. *Y*-axis showed the difference of ACC between the imputed and the observed datasets. ACCs of each observed dataset are shown. *p* values were calculated using one-tailed paired *t* test. NS indicates “not significant.” **a** PCMB dataset, **b** RETINA dataset, **c** neurons of BRAIN_SPLiT, and **d** non-neurons of BRAIN_SPLiT. The following approaches failed due to “out-of-memory” error: scImpute on RETINA, VIPER, and scImpute on BRAIN_SPLiT. Box plots show the median (center line), interquartile range (hinges), and 1.5 times the interquartile range (whiskers)

For the PMBC dataset, DISC (ACC = 0.91) and scImpute (ACC = 0.91) were the only approaches that have improved accuracy compared to the observed (ACC = 0.83). DISC had significantly better improvements compared to the other approaches except for scImpute (Fig. 5a). MAGIC and all the other four deep learning approaches, DeepImpute, DCA, scScope, and scVI, significantly dropped the classification accuracy compared to the observed (*p* value < 2.2e−16, one-tailed paired *t* test). Zooming into eight cell types, DISC achieved the top accuracy for all the cell types among all the approaches (Additional file 1: Fig. S5A). MAGIC failed identifying cell types using known marker genes due to the loss of marker genes for almost all the cell types.

For the RETINA dataset, DISC had the top performance and improved ACC from 0.83 (the observed) to 0.95 (Fig. 5b). Some rare cell populations, such as RGC, Muller glia, and VE, completely missed in the observed data due to dropouts, were recovered by DISC (Additional file 1: Fig. S5B). DISC performed significantly better than all the other approaches (*p* values shown in Fig. 5b). Although DCA improved the overall accuracy (ACC = 0.87), it mostly improved the identification of the major population, Rods, that counts for 66% of the total cell populations and completely missed identification of six other cell types. scScope only identified Rods and almost failed to identify all the other cell types while DeepImpute and VIPER completely missed identifying Rods. DISC had the top accuracy for 10 out of 11 cell types among all the approaches (Additional file 1: Fig. S5B).

The BRAIN_SPLiT dataset has 156,049 cells and we analyzed the cell types in neurons and non-neurons separately [3]. Because this dataset was sparse, with just 1329 mRNA counts per cell on average, and contained complex cell types, ACC score of neurons dropped from 0.48 to 0.2, and that of non-neurons dropped from 0.64 to 0.17 after down-sampling to 30%. Impressively, DISC improved ACCs to 0.46 and 0.58 for neurons and non-neurons after imputation (Fig. 5c, d). Four cell types in neurons and six cell types in non-neurons, including major cell types such as astrocyte and rare cell types such as Epend, missed due to dropouts after down-sampling, were recovered by DISC. DeepImpute and scScope almost completely failed to identify any cell types (Additional file 1: Fig. S5C and S5D).

We also compared all the approaches after down-sampling to 50% of the original datasets. The performance of DISC was consistent with the above analysis, indicating that DISC was robust to different dropout levels (Additional file 1: Fig. S6). In addition to ACC, adjusted rand index (ARI) was also used to evaluate the accuracy of cell-type classification. DISC also had the best accuracy for all the datasets (Additional file 1: Fig. S7). To sum up, DISC was the only approach consistently and significantly improved the accuracy of cell-type identification for all the datasets. DISC not only improved identification for both major and rare cell types, but also had robust performance on datasets generated from different single-cell platforms.

### DISC improves downstream analysis

We evaluated whether better gene expression structures translate to better results of downstream analysis. We evaluated similarities (1) between imputed scRNA-seq data and bulk RNA-seq data and (2) between DEGs identified by scRNA-seq data and bulk RNA-seq data, and (3) between pseudo-temporal order inferred by trajectory analysis and known cell differentiation order. Here, we used three datasets from 10X Genomics platform for this comparison (Methods).

Firstly, we calculated Spearman’s correlation coefficient (SCC) between the imputed scRNA-seq profiles and the bulk RNA-seq profiles for the same cell line and for the expression difference between two cell lines. All the imputation methods preserved the correlation between scRNA-seq profiles and bulk RNA-seq profiles (Additional file 1: Fig. S8A). But, only four methods preserved the correlation between the expression difference across the two cell lines of scRNA-seq profile and that of bulk RNA-seq profile, while DISC had the greatest improvement, improving 0.584 of RAW to 0.611 (Additional file 1: Fig. S8B) indicating the ability of DISC to capture the expression difference between cell types.

**Fig. 6:**
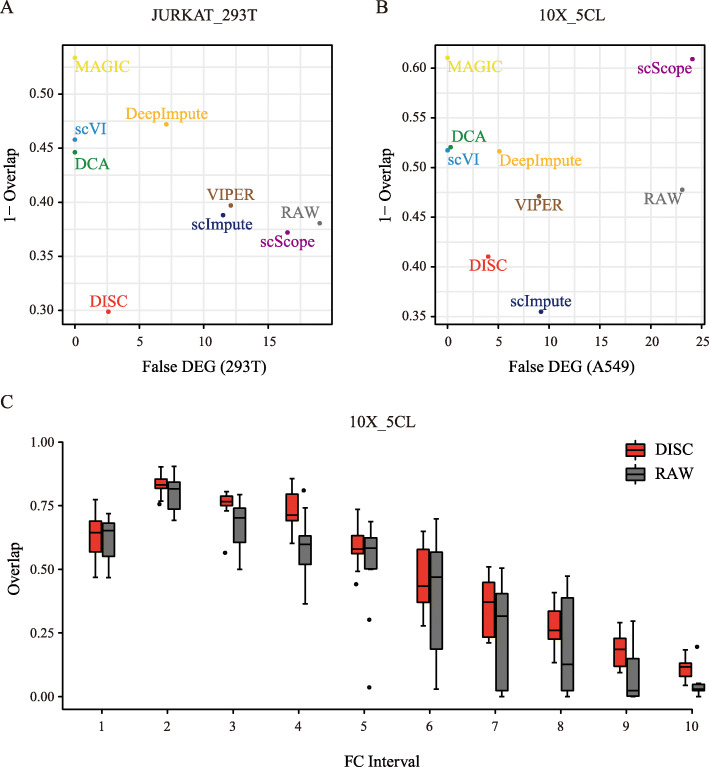
Evaluation of DEG identification. DEG was identified using MAST for **a** the JURKAT_293T dataset and **b** the 10X_5CL dataset, where *x*-axis shows the false number of DEGs identified from a homogeneous population (293T and A549 cell lines were used, respectively) and *y*-axis shows the averaged overlaps of DEGs identified by scRNA-seq data to that of bulk RNA-seq for all the combination of cell lines in the dataset. **c** For a pair of cell lines in the 10X_5CL dataset, genes were grouped into 10 intervals ranked by their FC values. For example, interval 1 is top 10% genes ranked by FC. The overlap between bulk and single-cell DEGs identified using MAST were calculated for 10 combinations of cell lines for each interval. Box plots show the median (center line), interquartile range (hinges), and 1.5 times the interquartile range (whiskers) for the 10 overlap values

We next evaluated DEG identification after imputation using DEGs identified by bulk RNA-seq data [19]. We used two methods, namely MAST [20] and Wilcoxon rank-sum test [21] (abbreviated as Wilcoxon), to identify DEGs for single-cell data. To evaluate the overall performance of DEG identification, we used two metrics, (1) the overlap of DEGs identified from the two cell types between the bulk data and scRNA-seq data and (2) the number of false detected DEGs using cells from a homogeneous population. DEGs identified by DISC using MAST had the first and second highest overlap to bulk for the two datasets, demonstrating the ability of DISC to improve DEG identification over RAW (Fig. 6a, b). Using Wilcoxon, DISC performed best for both datasets (Additional file 1: Fig. S9). At the same time, DISC was able to considerably decrease the number of false DEGs compared to the RAW dataset (Fig. 6a, b and Additional file 1: Fig. S9). Overall, DISC achieved a balance between sensitivity and specificity for DEG identification.

We then evaluated the impact of the magnitude of expression difference by fold-change (FC) [15]. For a pair of cell types in the 10X_5CL dataset, genes were ordered by their FC and then grouped into ten equal length intervals (each has 1815 genes). DISC showed improvement of the DEG overlaps between scRNA-seq data and bulk RNA-seq data for 7 out of 10 intervals while the overlaps retained or slightly decreased for the other 3 intervals, indicating a consistent improvement of DEG identification by DISC over RAW for different FCs (Fig. 6c). Comparing all methods, DISC performed well for the middle to the high FC intervals and performed moderately in low FC intervals, using both MAST (Additional file 1: Fig. S10A) and Wilcoxon (Additional file 1: Fig. S10B).

Finally, we evaluated the trajectory analysis. Different from the evaluation of DEG identification, cells in BONE_MARROW dataset were unsorted. Hence, we firstly identified cell types by mapping each cell in BONE_MARROW to a bulk cell type with the highest Spearman correlation [15]. Then, Monocle 2 were used to construct pseudo-temporal order for cells in BONE_MARROW dataset. Using the known differentiation order as the reference, the percentage of correctly ordered cell-pairs was used as the metrics for comparison. Consistent with the previous study [15], DCA, scVI, and MAGIC showed significant improvement of the inferred trajectory compared to RAW and DISC ranked third which is very close to the second scVI (Additional file 1: Fig. S11). In addition, the average distance between the mis-ordered cell-pairs was reduced by DISC to 1.65 from 2.31 of RAW (DISC was ranked the second and close to the best score of DCA (1.61). To sum up, DISC consistently improved the downstream analysis compared to the unimputed dataset and provided more biological meaningful information.

### DISC reliably identifies cell populations in the 1.3 million mouse brain dataset

**Fig. 7:**
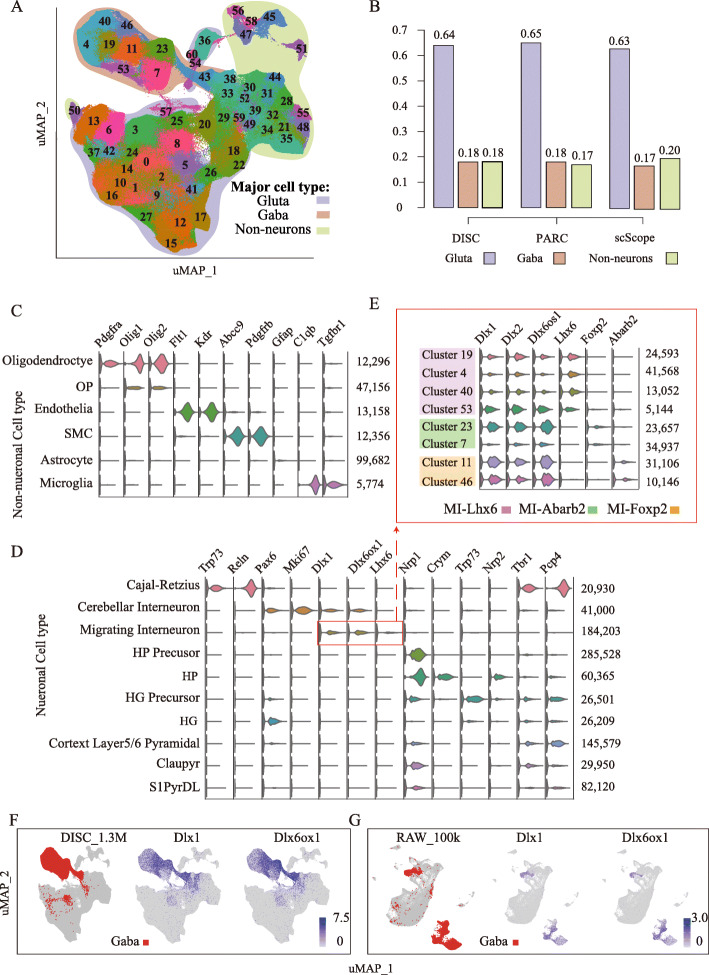
Analysis of BRAIN_1.3 M. **a** uMAP visualization using 50 compressed dimensions for 61 clusters identified by DISC. Clusters are split into three main cell types: glutamatergic neurons (Gluta), GABAergic neurons (Gaba), and non-neuronal cells. **b** The proportions of three main cell types identified by DISC, PARC, and scScope. **c**, **d** Cell types and marker genes for the non-neuronal cells (**c**) and the neuronal cells (**d**), the number of cells in each cell type is shown on the right. **e** Three sub-cell types and marker genes for MI. **f**, **g** Visualization of Gaba and MI marker genes, Dlx1 and Dlx6ox1, identified by DISC (**f**) and identified by Seurat (**g**) on 100,000 down-sampling cells

We finally analyzed the BRAIN_1.3 M dataset which was generated from multiple brain regions, including the cortex, hippocampus, and subventricular zone. In total, DISC identified 61 cell clusters (Fig. 7a and Additional file 1: Fig. S12). We assigned each cluster to one of three major cell groups, Glutamatergic neurons, GABAergic neurons and non-neuronal cells, using the known marker genes from the Allen Brain Atlas (Methods, Additional file 1: Table S5), which was also used by scScope and PARC [10, 22]. Approximately 1.1 million cells from 49 clusters were assigned to known cell types. The proportions of three main cell types are 64% for the Glutamatergic, 18% for the GABAergic, and 18% for the non-neuronal, which more closely agree with the composition reported by PARC (65, 18, and 17%) than scScope (63, 17, and 20%) (Fig. 7b). We assigned cells into 10 major neuronal (Fig. 7c) and 6 major non-neuronal cell populations (Fig. 7d); the marker gene used for cell types is shown in Additional file 1: Table S6. The smallest cell population is Microglia (5774 cells), which had unique cell markers of C1qb and Tgfbr1, counting for 0.44% cells of the dataset (Fig. 7c). These cell populations can be further categorized into sub-cell populations. For example, migrating interneurons (MI) can be further sub-grouped into three sub-populations based on distinguishing sub-cell markers (Fig. 7e).

Compared to the cell types identified by DISC and scScope, we found a large discrepancy from MI. DISC identified 184,203 MI cells (14.36%) belonging to GABAergic neurons (Fig. 7d), while scScope identified 543,779 MI cells (42.40%) belonging to glutamatergic neurons. By visualizing two MI markers, Dlx1 and Dlx6os1, our analysis clearly showed that MI belonged to GABAergic groups (Fig. 7f). To confirm our result, we used a commonly used cell-type identification tool, Seurat. Because Seurat was not able to handle such a large dataset, we down-sampled 100,000 out of 1.3 million cells. Consistent with our analysis, the analysis by Seurat also showed a clear signal that MI belonged to GABAergic neurons, accounting for 14% in 100,000 cells (Fig. 7g). These results demonstrate DISC’s ability to efficiently and accurately explore the major and rare cell populations in ultra-large heterogeneous single-cell datasets.

## Discussion

Many factors such as expression level and distribution, level of noises, and heterogeneity of cells affect the performance of imputation. DISC assumes no specific distribution of expression and dropouts. Semi-supervised deep learning framework allows DISC to learn a complex structure of genes and cells from sparse data. Unlike the other imputation approaches, DISC does not down-sample genes for the model input therefore preserves the more information from the data. As a result, DISC showed robust performance to datasets with different sizes, different dropout levels, and from different platforms. We expect that DISC will continue working well as the noise distribution changes with the emerging novel platforms of scRNA-seq.

Although DISC and scScope have similar network structures, they are trained by different strategies. DISC employs semi-supervised learning and its loss function is computed on both positive-count genes (real labels) and zero-count genes (pseudo labels), while scScope is trained in a supervised-manner and its loss function is computed only on positive-count genes [10]. When positive-count genes are limited, training based only on positive-count genes is likely to miss the distinguishing features between technical and biological dropouts where both belong to zero-count genes, thus leading to a latent representation that best interprets positive-count genes without properly encodes distinguishing features for dropouts. Semi-supervised learning supplements the training with zero-count genes to build a more reasonable latent representation which also best interprets zero-count genes structurally similarly to positive-count genes as a technical dropout. As a consequence, DISC distinguishes the technical zero generated by down-sampling (Additional file 1: Fig. S13A and S14A) and scScope was only able to distinguish technical zero generated by down-sampling in RETINA and BRAIN_SPLiT datasets (Additional file 1: Fig. S13B and S14B), which contain a large number of cells and positive-count genes. Hence, DISC works well when the information provided by positive-count genes is limited.

Some recent studies concerned biases introduced by imputation [13, 14]. In our study, we also found that several imputation approaches not only considerably changed genetic and cellular structures of scRNA-seq data, but also significantly decreased the accuracy of downstream analysis, such as identification of DEGs and cell types, after imputation. In contrast, we demonstrated that DISC not only recovered gene expression and enhanced gene structures, but also significantly improved the accuracy of downstream analysis. Compared to the other seven approaches, DISC consistently achieved top performance on ten real-world datasets using various evaluation metrics, illustrating its robust and stable performance.

In the last few years, advances in scRNA-seq technology have enabled us to obtain a few thousands to over a million of cells in just one study. Moreover, the integration of datasets from different studies could provide much more biological insights than the single study does [23, 24]. It is therefore an urgent task to establish an analytic method capable of handling ultra-large datasets. We showed that DISC could readily handle over several million of cells using just a small portion of computational cost that other deep learning-based approaches used. Unlike other imputation approaches, DISC processed large datasets with tens of thousands of genes, which minimizes information lost due to gene or cell down-sampling.

## Conclusions

In conclusion, our results demonstrated that DISC should be used for imputation, particularly for datasets with sparse expressed genes. Making no assumption to data distribution, DISC provides a general solution for analyzing single-cell omics data. It outputs both expression matrix and low dimensional representations, which can be used for clustering and visualization by other analytical tools that have no capability to deal with ultra-large datasets. We expect that DISC will be of immediate interest to the fast-growing single-cell research community.

## Methods

### Description of DISC

#### Normalization

The cell expression profile of cell *c* with *M* genes in mRNA counts *C*_*c*_ ∈ ℤ^*M*^ is firstly normalized by cell library size with log transformation

$$ \tilde{C_c}=\ln \left( sf\frac{C_c}{ls_c}+1\right), $$

where *ls*_*c*_ is the library size of cell *c*, and *sf* (scale factor) is a constant, defined below.

#### Outlier detection

We use a scale factor of 1 million for normalization and calculate *Z* scores for the normalized counts over all cells. Genes with *Z* scores greater than three are treated as outliers. DISC does not impute outliers so that outliers stay unchanged in the imputed expression matrix. During training, DISC masks outliers and uses semi-supervised learning framework to assign pseudo-counts for the outliers for training.

#### Input preparation

We use a scale factor of median cell library size for normalization and scale each gene *m* (1 ≤ *m* ≤ *M*) by its normalized max (excluding outliers) over all cells to 0–1.

$$ {x}_{c,m}^0=\frac{\overset{\sim }{C_{c,m}}}{\overset{\sim }{C_{\max, m}}}, $$

where $\left\{{x}_c^0\in {\mathrm{\mathbb{R}}}^M|0\le {x}_{c,m}^0\le 1\right\}$ is the first step input and RNN will repeats for *T* steps.

#### Encoder

The encoder layer *f*_*E*_(⋅) projects input of step *t*${x}_c^t$ into a low-dimensional, latent representation ${z}_c^t\in {\mathrm{\mathbb{R}}}^S,S<M$. The encoder layer is given by

$$ {z}_c^t={f}_E\left({x}_c^t\right)=\tanh \left({w}_E{x}_c^t\right), $$

where *w*_*E*_ is a learnable parameter.

#### Decode

In contrast to the encoder layer, decoder layers *f*_*D*_(⋅) reverses the latent representation back into a reconstructed normalized expression profile $\left\{\hat{y_c^t}\in {\mathrm{\mathbb{R}}}^{\mathrm{M}}|0\le \hat{y_{c,m}^t}\le 1\right\}$, given by

$$ \hat{y_c^t}={f}_D\left({z}_c^t\right)=\mathrm{sigmoid}\left(2\left(\varphi +1\right)\circ \left({w_E}^{\mathrm{T}}{z}_c^t+{b}_D\right)\right), $$

where *w*_*E*_^T^ is the transpose of *w*_*E*_ and *φ* ∈ ℝ^*M*^, *b*_*D*_ are learnable parameters.

#### Prediction matrix

The prediction matrix contains *M* channels, each channel *f*_*P*, *m*_(⋅) predicts the expression of a single gene $0\le {y}_{c,m}^t\le 1$ from the latent representation ${z}_c^t$ as

$$ {y}_{c,m}^t={f}_{P,m}\left({z}_c^t\right). $$

A channel has three layers, given by

- 1st hidden layer: $$ h{1}_{c,m}^t=\left({\varphi}_m+1\right)\left({w}_{h1,m}{z}_c^t+{b}_{h1,m}\right) $$,
- 2nd hidden layer: $$ h{2}_{c,m}^t=\left({\varphi}_m+1\right)\left({w}_{h2,m}\cdot \tanh \left(h{1}_{c,m}^t\right)+{b}_{h2,m}\right) $$,
- Output layer: $$ {y}_{c,m}^t=\mathrm{sigmod}\left(2\left({\varphi}_m+1\right)\left(\begin{array}{l}{\psi}_{c,m}^t\left({w}_{p1,m}\tanh \left(h{2}_{c,m}^t\right)+{b}_{p1,m}\right)\\ {}+\left(1\hbox{-} {\psi}_{c,m}^t\right)\left({w}_{p2,m}\tanh \left({h}_{2,m}^t\right)+{b}_{p2,m}\right)\end{array}\right)\right) $$, $$ {\psi}_{c,m}^t=\mathrm{sigmoid}\left(\mathrm{SELU}\left({w}_{\psi, m}h{2}_{c,m}^t\right)\right) $$,

where *w*_*h*1, *m*_, *w*_*h*2, *m*_, *w*_*p*1, *m*_, *w*_*p*2, *m*_, *w*_*ψ*, *m*_, *b*_*h*1, *m*_, *b*_*h*2, *m*_, *b*_*p*1, *m*_, and *b*_*p*2, *m*_ are learnable parameters for gene *m*. The output layer is a weighted average over two channels using ${\psi}_c^t$ as weight factor. We assumed that a given gene followed the same expression distribution across most of cells and defined this as “major expression distribution”. Before sigmoid activation, scaled exponential linear unit [25] (SELU) activation is used to make the channel selection biased the first channel, where the first channel represents the major expression distribution.

#### Filter

Input for the next step, ${x}_c^{t+1}$, is prepared by filtering of ${y}_c^t$ to keep the positive-counts as

$$ {x}_{c,m}^{t+1}=\left\{\begin{array}{c}{x}_{c,m}^0,\kern0.5em {x}_{c,m}^0>0\\ {}{y}_{c,m}^t,{x}_{c,m}^0=0\end{array}\right.. $$

#### Imputer and reconstructor

A soft attention assigns a weight vector ${a}_c^t$ to the decoding $\hat{y_c^t}$ and prediction ${y}_c^t$ output from each recurrence. ${a}_c^t$ is given by

$$ {a}_{c,m}^t=\mathrm{softmax}\left({w}_{a,m}\mathrm{SELU}\left(h{1}_{c,m}^t\right)\right), $$

where *w*_*a*, *m*_ is a learnable parameter. After weighted average, $\left\{\hat{y_c}\in {\mathrm{\mathbb{R}}}^{\mathrm{M}}|0\le \hat{y_{c,m}}\le 1\right\}$ and {*y*_*c*_ ∈ ℝ^M^| 0 ≤ *y*_*c*, *m*_ ≤ 1} are given by

$$ \hat{y_c}=\sum \limits_t{a}_c^t\circ \hat{y_c^t} $$ and $$ {y}_c=\sum \limits_t{a}_c^t\circ {y}_c^t $$.

#### Compressor

The latent representations over all steps, *z*_*c*_ ∈ ℝ^*S* × *T*^, are compressed further to a lower dimension *W* ≪ *S* ⋅ *T*. Compressor is an autoencoder whose encoder is given by

$$ {cp}_c=\tanh \left({w}_z{z}_c+{b}_{z1}\right), $$

And the reverse decoder is given by

$$ \tilde{z_c}=\tanh \left({w_z}^{\mathrm{T}}{cp}_c+{b}_{z2}\right), $$

where *w*_*z*_, *b*_*z*1_, and *b*_*z*2_ are learnable parameters, *cp*_*c*_ is the compressed cell feature where *cp*_*c*_ ∈ ℝ^*W*^. Autoencoder and compressor modules together form a stacked autoencoder. To evaluate the performance of the compressor, the cell expression profile $\tilde{y_c}$ is reversed from $\tilde{z_c}\in {\mathrm{\mathbb{R}}}^{S\times T}$, given by

$$ \tilde{y_c}=\sum \limits_t{a}_c^t\circ \tilde{y_c^t}, $$

where *a*^*t*^ is the shared soft attention weight for the imputer and reconstructor modules and $\tilde{y_c^t}$ is reversed from $\tilde{z_c^t}$ using the decoder module.

### Training of DISC

The parameters of DISC are optimized from the data in an end-to-end manner according to a combination of five loss functions, including imputation loss (*L*_*I*_), reconstruction loss (*L*_*R*_), prediction loss (*L*_*P*_), latent representation loss (*L*_*LR*_), and constraint (*L*_*C*_).

#### Imputation loss

*L*_*I*_ is formulated based on the idea of “noise to noise” for image imputation [18]. A noise input ${nx}_c^0$ for the first step is prepared by assigning an uniform multiplicative noise: ${U}_c^M\left(0.9,1.1\right)\circ {x}_c^0$ and ${nx}_c^0$ replaces ${x}_c^0$ for filtering of predicted expression profile to produce inputs for the later steps, ${nx}_c^t$. In addition, a dropout operation is applied to ${nx}_c^t$ on zero-count genes in raw data [26]. At the end, a noise imputation output *ny*_*c*_ is produced and *L*_*I*_ is formulated as

$$ {L}_I=\frac{1}{N}\sum \limits_c{\left\Vert \alpha {1}_c\circ \left({ny}_c-{ny_c}^{\hbox{'}}\right)\right\Vert}_1, $$

where *ny*_*c*_^'^ is a noise target given by ${U}_c^M\left(0.9,1.1\right)\circ {x}_c^0$ and *N* is the number of cells for training. *L*_*I*_ is computed on the positive-counts restricted by *α*1, given by

$$ \alpha {1}_{c,m}=\left\{\begin{array}{cc}1,& {x}_{c,m}^0>0\\ {}0,& {x}_{c,m}^0=0\end{array}\right.. $$

#### Reconstruction loss

*L*_*R*_ is formulated using semi-supervised learning (SSL) to learn a concordant latent representation which encodes both positive-counts and pseudo-counts assigned by the imputer as

$$ {L}_R=\frac{1}{N}\sum \limits_c{\left\Vert \alpha {2}_c\circ \left(\hat{y_c}-\hat{{y_c}^{\hbox{'}}}\right)\right\Vert}^2, $$

where $$ \alpha {2}_{c,m}=\left\{\begin{array}{cc}{\alpha}_R,& {x}_{c,m}^0>0\\ {}1,& {x}_{c,m}^0=0\end{array}\right. $$, *α*_*R*_ balances the biased portions towards zero-counts, the reconstruction target is $$ \hat{{y_{c,m}}^{\hbox{'}}}=\left\{\begin{array}{cc}{x}_{c,m}^0,& {x}_{c,m}^0>0\\ {}{y}_{c,m},& {x}_{c,m}^0=0\end{array}\right. $$.

#### Prediction loss

*L*_*P*_ uses SSL to search an expression profile structure which underlying both positive-counts and pseudo-counts assigned by the decoder, given by

$$ {L}_P=\frac{1}{N}\sum \limits_t\sum \limits_c{\left\Vert \alpha {3}_c\circ \left({y}_c^t-{y_c^t}^{\hbox{'}}\right)\right\Vert}^2, $$

where $$ \alpha {3}_{c,m}=\left\{\begin{array}{cc}{\alpha}_{P1},& {x}_{c,m}^0>0\\ {}{\alpha}_{P2},& {x}_{c,m}^0=0\end{array}\right. $$ and the prediction target is $$ {y_{c,m}^t}^{\hbox{'}}=\left\{\begin{array}{ll}{x}_{c,m}^0,& {x}_{c,m}^0>0\\ {}\hat{y_{c,m}^t},& {x}_{c,m}^0=0\end{array}\right. $$.

#### Latent representation loss

Prediction of expression profile made by each step is a function of the corresponding latent representation. *L*_*LR*_ minimizes the difference between successive latent representations, given by

$$ {L}_{LR}=\frac{1}{N\cdot T}\sum \limits_{t=1}^{\mathrm{T}+1}\sum \limits_c{\left\Vert {x}_c^t{w}_E-{x}_c^{t-1}{w}_E\right\Vert}^2. $$

#### Constraint

*L*_*C*_ limits the total capacity of imputation counts assuming most zero-counts are either low expressed or unexpressed. *L*_*C*_ is given by

$$ {L}_C=\sum \limits_t\sum \limits_c{\left\Vert \alpha {4}_c\circ {f}_{de}\left({y}_c^t\right)\right\Vert}^2, $$

where $$ \alpha {4}_{c,m}=\left\{\begin{array}{cc}0,& {x}_{c,m}^0>0\\ {}1,& {x}_{c,m}^0=0\end{array}\right. $$ and *f*_*de*_(⋅) is a function reverses the normalized counts back to counts.

#### Regularization

We assumed that some genes contribute more (strong connection) to each neuron of the latent representation. However, conventional sparse regularizers, i.e., L1 regularizer and Log regularizer, are unable to restrict the number of genes having strong connections to the neurons. We developed a new regularizer, *f*_*re*_, to restrict the genes as

$$ {f}_{re}(w)=\sum \limits_i^{NN_w}{\left(\sum \limits_{j\in {w}_i}{j}^2\right)}^2, $$

where *NN*_*w*_ is the number of output-nodes, *w*_*i*_ is the collection of weights connecting with *i*th output-node. *j*^2^ removes weights that are very small.

The overall loss function is

$$ {\displaystyle \begin{array}{l}\mathrm{L}={\beta}_1{L}_I+{\beta}_2{L}_R+{\beta}_3{L}_P+{\beta}_4{L}_{LR}+{\beta}_5{L}_C\\ {}\kern1.25em +{\beta}_6\left({f}_{re}\left({w}_E\right)+{f}_{re}\left({w}_{h1}\right)\right)\\ {}\kern1.5em +{\beta}_7\sum \limits_{w\in {w}_{h2},{w}_{p_1},{w}_{p_2}}{w}^2+{\beta}_8\sum \limits_{w\in {w}_a}{w}^2+{\beta}_9\sum \limits_{w\in \varphi }{w}^2.\end{array}} $$

DISC was trained using Adam [27] with learning rate 0.001. Gradient clipping of 5 was used to avoid exploding gradient.

#### Stop of training

Predictor of DISC is a function of the latent representation, *z*^*t*^. When the difference of *z*^*t*^ across multiple steps becomes smaller, DISC is convergent to a stable point. Therefore, DISC uses latent representation loss to evaluate the similarity of *z*^*t*^ across multiple steps and to determine the best stop point based on the variance of this loss over multiple batches (10,000 batches by default). We chose 5 million cells as an initial point because DISC generally reached optimal points after learning information from approximately 5 million cells in many datasets with a variety of gene and cell numbers. This property makes DISC a stable running time for datasets of various sizes. The procedure is as follows.

1. DISC is first trained for 5 million cells (128 cells per training batch on default) and calculates the standard deviation (STD) of *L*_*LR*_ for the last 10,000 batches. This STD is set as the minimum STD, and this STD remains as the minimum STD for 1 round (minimum round where a training round is 50,000 cells).
2. DISC is trained for another 50,000 cells and calculates a new STD of *L*_*LR*_ for the last 10,000 batches.
3. If the new STD is greater than the minimum STD, minimum round is increased by 1. Otherwise, minimum STD is set as the new STD, and minimum round is reset to 1.
4. If minimum round is less than 5, repeat step 2. Otherwise, training is stopped.

### Hyperparameter optimization

Hyperparameters for the model architecture, including layer neuron numbers, number of steps and learning rate, are pre-defined (Additional file 1: Fig. S1), and the other ones were sampled using Latin hypercube sampling [28]. We provided a set of hyperparameters as the default so that users can easily use DISC without to undergo the time-consuming optimization process. We tested the default hyperparameter set for many high-throughput single cell datasets with different cell numbers (thousands to millions), different platforms, and different cell compositions, and the performance was robust. The following hyperparameters are set as the default value:

*α*_*R*_ = 5, *α*_*P*1_ = 1.5, *α*_*P*2_ = 0.35, *β*_1_ = 1, *β*_2_ = 1, *β*_3_ = 1, *β*_4_ = 1.65 × *M* × 1*e*^−5^, *β*_5_ = 6.3 × 1*e*^−5^, *β*_6_ = 1*e*^−6^, *β*_7_ = 1*e*^−6^, *β*_8_ = 1*e*^−5^, and *β*_9_ = 1*e*^−4^. Users are also able to change them via command line interface.

### Generating training batches

To randomize cell orders in an expression matrix, a common practice is to load a complete expression matrix into memory and random sample cell batches. However, loading large expression matrix usually causes out-of-memory errors (OOM). A previous method split the expression matrix into several parts and saved onto hard disk [10]. During training, parts were loaded separately to generate random cell batches. However, by this approach, random sampling was performed locally within the parts and pre-processing required extra work. Here, we developed a novel method to generate globally random cell batches.

1. Cells are indexed by chunks of arbitrary size (32 cells by default).
2. Multiple chunks are loaded randomly (64 chunks by default) into a sub-queue in the memory and cells in the sub-queue are shuffled. Once shuffled, cells are transferred into a main queue in the main thread.
3. Cells are loaded parallelly via parallel sub-queues to reduce the loading delay and cells from different sub-queues are transferred randomly into the main queue.
4. At the end, cell batches are withdrawn from the main queue based on first-in-first-out rule.

### Cell-type identification

#### Small and large datasets

For smaller datasets, including PBMC, RETINA, neuronal cells (129 K cells), and non-neuronal cells (27 K cells) of BRAIN_SPLiT datasets, Seurat V3.0 [29] was used to perform normalization, feature selection, scaling, PCA, clustering, and t-SNE/u-MAP visualization. Resolution and PCA-dimension parameters for clustering were selected to produce the best accuracy against cell-type labels. Specifically, resolution of 0.5–1.4 (0.5 for PBMC, 1.4 for RETINA, 1.4 for BRAIN_SPLiT) and top 10–50 principal components of PCA (10 for PBMC, 30 for RETINA, 50 for BRAIN_SPLiT) were used and clustering was based on the graph-based shared nearest neighbor method (SNN). Differential expression analysis was used to identify cluster-specific marker genes where all the clusters are pairwise compared using the Wilcoxon method. Each identified marker gene was expressed in a minimum of 25% of cells and at a minimum log fold change threshold of 0.25. When the cluster-specific marker genes contain the reference cell type, we defined the cluster as the reference cell type. However, if multiple reference marker genes for different cell types or no reference marker genes appeared in the cluster-specific marker genes, we defined these clusters as unknown cell types.

#### Ultra-large dataset

For the BRAIN_1.3 M dataset with 1.3 million cells, traditional methods are unable to cluster cells using the whole expression matrix. Compressed features of 50 dimensions from DISC were used for clustering by Seurat, where the resolution was set to 1.4. Differential expression analysis was described above.

### Evaluation of imputation performance

#### Gene selection

Genes match the following conditions were removed:

1. Expressed in less than 1/1000 of total cells or less than 10 cells, whichever is greater.
2. Maximum mRNA count is 1.

#### Comparison of scRNA-seq and FISH

Genes overlapped between scRNA-seq (≥ 10 positive-count cells) and FISH were selected. To compare the expression distributions of scRNA-seq and FISH, each selected gene was normalized by an efficient factor [6], where efficient factor was defined as the ratio of its FISH mean to its scRNA-seq (raw or imputation) mean.

#### Down-sampling

We randomly sampled transcript reads from scRNA-seq dataset followed a previous research [5]. Transcripts were sampled either 30 or 50% of the original cell library size.

#### Gini coefficient

We used “reldist” package in R to calculate Gini coefficient to quantify gene expression distribution [30]. The difference of Gini coefficients between scRNA-seq (raw and imputation) and FISH was calculated by rooted mean square error (RMSE), given as

$$ \mathrm{Gini}\ \mathrm{RMS}{\mathrm{E}}_{\mathrm{method}}=\sqrt{\frac{\sum \limits_{i=1}^n{\left({\mathrm{Gini}}_{\mathrm{FISH},i}-{\mathrm{Gini}}_{\mathrm{method},i}\right)}^2}{n}}, $$

where *n* is the number of overlapped genes, *i* is the index of the genes.

#### Fasano and Franceschini’s test

Kolmogorov-Smirnov (K-S) distance [31] is a nonparametric estimation of the distance between two one-dimensional probability distributions, based on their cumulative distributions. Fasano and Franceschini’s (FF) distance [32] is a multi-dimensional version of K-S distance. Using FISH data as the reference, we used a script (https://github.com/syrte/ndtest/blob/master/ndtest.py) to calculate FF distance as a measurement for the similarity of the gene-gene co-expression distribution between scRNA-seq (raw and imputation) and FISH.

#### Correlation matrix distance (CMD)

CMD is a measure of the distance between two correlation matrices [33]. The CMD for two correlation matrices *R*_1_, *R*_2_ is defined as

$$ d\left({R}_1,{R}_2\right)=1-\frac{tr\left({R}_1,{R}_2\right)}{{\left\Vert {R}_1\right\Vert}_f{\left\Vert {R}_2\right\Vert}_f}. $$

For comparison with FISH, Pearson’s correlation was calculated for gene pairs in *R*_1_ (FISH) and *R*_2_ (raw or imputation) using all the overlapped genes. For comparison in down-sampling dataset, Pearson’s correlation was calculated for gene pairs in *R*_1_ (reference) and *R*_2_ (observed or imputation) using the top 300 variable feature genes selected by Seurat’s “vst” function [23, 29].

#### Mean absolute error (MAE)

MAE measures the difference of gene expressions of the observed or imputation data to the reference data, given by

$$ {\mathrm{MAE}}_c=\frac{\sum \limits_{i={D}_c}^n\left|{C}_{c,i}^{ds}\times {sf}_c-{C}_{c,i}^{\mathrm{reference}}\right|}{\operatorname{card}\left({D}_c\right)}, $$

where *D*_*c*_ is the set of positive-count genes in cell c from the reference data, card(*D*_*c*_) is the size of set *D*_*c*_, *C*^*ds*^ is the observed/imputed mRNA counts, *C*^reference^ is the mRNA counts before down-sampling, and ${sf}_c={ls}_c^{\mathrm{reference}}/{ls}_c^{ds}$, where *ls*^reference^ and *ls*^*ds*^ are the cell library size vectors for the corresponding datasets.

#### Gene-gene and cell-cell correlation

Pearson’s correlation was calculated at the gene or cell levels before and after down-sampling. At the gene level, genes were included if they express in at least 10% of cells. At the cell level, cells were included if they have at least 10% of gene expressed.

#### Evaluation of cell-type annotation accuracy

To evaluate cell-type accuracy, three evaluation metrics are used. Accuracy (ACC) and adjusted rand index (ARI) are used to assess the properties of the overall clustering results and Jaccard index is used to calculate the accuracy of each cell type.

ACC is calculated as

$$ \mathrm{ACC}=\frac{\sum \limits_{i=1}^n\delta \left({r}_i,{s}_i\right)}{n} $$

where *n* is the cell number, *r*_*i*_ and *s*_*i*_ are the cell type label and classified cell type, respectively, for *i*th cell, and

$$ \delta \left(x,y\right)=\left\{\begin{array}{cc}1& \mathrm{if}\kern0.5em x=y\\ {}0& \mathrm{otherwise}\end{array}\right.. $$

The overlap between the cell type labels and classified cell type can be summarized in a contingency table, in which each entry denotes the number of objects in common between the two sets.

ARI is calculated as

$$ \mathrm{ARI}=\frac{\sum \limits_{i=1}^{\left|\mathrm{K}\right|}\sum \limits_{j=1}^{\left|\mathrm{K}\right|}\left(\begin{array}{c}{n}_{i,j}\\ {}2\end{array}\right)-\left[\sum \limits_{i=1}^{\left|\mathrm{K}\right|}\left(\begin{array}{c}{a}_i\\ {}2\end{array}\right)\sum \limits_{j=1}^{\left|\mathrm{K}\right|}\left(\begin{array}{c}{b}_j\\ {}2\end{array}\right)\right]/\left(\begin{array}{c}n\\ {}2\end{array}\right)}{\frac{1}{2}\left[\sum \limits_{i=1}^{\left|\mathrm{K}\right|}\left(\begin{array}{c}{a}_i\\ {}2\end{array}\right)\sum \limits_{j=1}^{\left|\mathrm{K}\right|}\left(\begin{array}{c}{b}_j\\ {}2\end{array}\right)\right]-\left[\sum \limits_{i=1}^{\left|\mathrm{K}\right|}\left(\begin{array}{c}{a}_i\\ {}2\end{array}\right)\sum \limits_{j=1}^{\left|\mathrm{K}\right|}\left(\begin{array}{c}{b}_j\\ {}2\end{array}\right)\right]/\left(\begin{array}{c}n\\ {}2\end{array}\right)}, $$

where K is the set of unique cell type labels, *n*_*i*, *j*_ are values from the contingency table, *a*_*i*_ is the sum of the *i*th row of the contingency table, *b*_*j*_ is the sum of the *j*th column of the contingency table, and ( ) denotes a binomial coefficient and $$ \left(\begin{array}{c}n\\ {}2\end{array}\right) $$ means $\frac{n\left(n-1\right)}{2}$.

Jaccard index is calculated as

$$ J\left(c,d,k\right)=\frac{\left|c\cap d\right|}{\left|c\right|+\left|d\right|-\left|c\cap d\right|}, $$

respectively, where *c* is the set of cells with type labels *k*, *d* is the set of cells with classified cell type, and *k* ∈ Κ.

#### Ranking differentially expressed genes (DEGs)

For the bulk RNA-seq samples, DEGs were identified using the limma R/Bioconductor package. We corrected *p* values for multiple testing using the Benjamini-Hochberg (BH) method (p.adjust function in the stats R package) to derive false discovery rate (FDR). Genes with FDR smaller than *α* = 0.05 and log fold change greater than 1.5 were identified as DEG and used as the “gold standard” in the following comparison. The number of DEGs identified is shown in Additional file 1: Table S7. For the scRNA-seq data, DEGs were identified using MAST and (2) Wilcoxon rank-sum test, where we used Seurat FindMarkers function to perform the two methods, and set min.pct = 0.1, logfc.threshold = 0 to get the difference of all genes. The single-cell DEGs were ranked by p_val_adj or the log-scaled expression fold change if there was a tie for p_val_adj. For *i* from 1 to *k*, we calculated the proportion of top 10 * *i* single-cell DEGs that overlap with bulk DEGs while the average of these *k* proportions served as the performance metric. For comparing DEG identification from all genes, *k* was set at 100, and for comparing DEG identification in 10 intervals sorted by FC, *k* was set at 10.

#### Null differential analysis

There are no DEGs in a homogeneous population of cells, such as 293T cells from the JURKAT_293T dataset (*N* = 2885 cells) and A549 cells from the 10X_5CL dataset (*N* = 1256 cells). Therefore, for each dataset, we randomly sampled cells into two groups with group sizes ranging from *N* = 10 to 500 [15]. We conducted DEG analysis for 10 conditions with different cell numbers (10 vs. 10, 10 vs. 50, 10 vs. 100, 10 vs.500, 50 vs. 50, 50 vs.100, 50 vs. 500, 100 vs. 100, 100 vs. 500, 500 vs. 500). We identified DEGs using MAST and Wilcoxon rank-sum test. Genes with *p* value < 0.01 and logfc > 0.25 were identified as DEG.

#### Trajectory analysis

Following a previous study [15], the bulk-sequencing data from 13 hematopoietic cell types of 3 cell lineages, lymphoid, erythroid, and myeloid (GSE74246) were used to identify hematopoietic cells profiled by 10X Genomics single-cell platform (BONE_MARROW). Briefly, each cell in BONE_MARROW was marked as one of the 13 hematopoietic cell types whose bulk RNA-seq profile has the highest Spearman’s correlation with the cell’s scRNA-seq profile. Then, the scRNA-seq expression matrices were input into Monocle2 to construct pseudo-temporal trajectories using DDRTree algorithm. The known differentiation levels (HSC: level 1; MPP: level 2; LMPP and CMP: level 3; CLP, GMP, and MEP: level 4; B cell, CD4 T cell, CD8 T cell, NK cell, Monocyte, and Erythroid: level 5) served as the reference to compare the correctness of the order inferred between cell-pair from two different differentiation level cell types which are both appeared in at least one of the lymphoid, erythroid, and myeloid lineages. If a cell has a higher differentiation level in the pair, then the cell must have a higher pseudo-time to define the pair in a correct order. For example, for a pair of cells, a HSC and a Monocyte, the order of the pair is inferred correctly if the Monocyte is marked a higher pseudo-time than the HSC since the Monocyte has a higher differentiation level than the HSC. Since the root state can be any of the leaves of the constructed trajectories tree and the root state determines the pseudo-time for all the cells (root state is the pseudo-time 0). Hence, the overall percentage of correctly inferred cell pairs for all the possible cell pairs was calculated for all possible root state and the highest percentage was used for comparison.

### Comparison of imputation approaches

The imputation approaches were run on a Linux CentOS 7 server with 2 Intel® Xeon® E5–2650 v4 CPUs, 128GB RAM and 1 NVIDIA® Tesla® V100 GPU. Unless otherwise noted, software packages were used with their default settings after gene selection. For all deep learning methods (DISC, DCA, scVI and scScope), GPU were used for training and imputation. The running scripts can be found at https://github.com/xie-lab/DISC/tree/master/reproducibility/source/Running%20Scripts%20for%20Other%20Methods.

#### Speed and memory comparison

Speed and memory usage were compared using BRAIN_1.3 M dataset. Cells express less than 500 or greater than 5000 genes were removed (approximately 1.3 million cells left). The top 1000 or 10,000 highly variable genes were selected using “vst” (variance stabilizing transformation) of Seurat. We then randomly sampled 3 subsets in different cell numbers (50 k, 100 k and 500 k cells). We duplicated 1.3 M datasets into a 2.6 M cell dataset. For each imputation method compared, we ran each dataset 3 times and calculated the average computation time and memory usage.

#### Methods comparisons

##### Magic

We used the Python package of magic-impute v1.5.5. Following its tutorial (https://nbviewer.jupyter.org/github/KrishnaswamyLab/MAGIC/blob/master/python/tutorial_notebooks/emt_tutorial.ipynb), we performed library size normalization and square root transformation before imputation. We then squared and denormalized its output gene expressions after imputation.

##### scImpute

We used the R package of scImpute v0.0.9.

##### VIPER

We used the R package of VIPER (GitHub commit 0170c27). Following its README (https://github.com/ChenMengjie/VIPER/blob/master/README.md), we used its gene-based imputation.

##### DCA

We used the Python package of DCA v0.2.2.

##### scScope

We used the Python package of scScope v0.1.5. Following its demo script (https://github.com/AltschulerWu-Lab/scScope/blob/master/demo.py), we normalized each cell to have the same library size, set the feature dimension as 50 and then imputed dropout values after training with the default setting.

##### scVI

We used the Python package of scVI v0.3.0, followed the reproducibility script (https://github.com/YosefLab/scVI/blob/aa614bdaf2ff57fbb661394e53a9a2454b950882/tests/notebooks/scVI_reproducibility.ipynb).

##### DeepImpute

We used the Python package of deepImpute v1.0.0.

### Availability of data and materials

#### MELANOMA (GSE99330, 8498 melanoma cells by Drop-seq) with FISH

Eight thousand six hundred forty cells from the melanoma WM989 cell line were sequenced using Drop-seq [34], where 32,287 genes were detected. Eight thousand four hundred ninety-eight cells were extracted according to the previous pipeline [6] and 15,204 genes were left after gene selection. In addition, RNA FISH experiment of across 7000–88,000 melanoma cells from the same cell line was conducted and 26 were detected [35], in which 19 genes were overlapped with the 15,204 genes, including 9 housekeeping genes (*BABAM1*, *GAPDH*, *LMNA*, *CCNA2*, *KDM5A*, *KDM5B*, *MITF*, *SOX10*, and *VGF*) and 10 drug-resistance markers (*C1S*, *FGFR1*, *FOSL1*, *JUN*, *RUNX2*, *TXNRD1*, *WNT5A*, *EGFR*, *PDGFC*, and *VCL*). RNA-seq data can be found at GSE99330. RNA FISH data can be found at https://www.dropbox.com/s/ia9x0iom6dwueix/fishSubset.txt?dl=0.

#### SSCORTEX (SRP135960, 3447 and 3969 mouse somatosensory cortex cells in 2 replications by 10X Genomics) with FISH

Mouse somatosensory cortex of CD-1 mice at age of p28 and p29 were profiled by 10X where 7477 cells were detected in total [36]. Cells expressed less than 500 or greater than 5000 genes were removed (7416 cells left) and 13,997 genes were left after gene selection. osmFISH experiment of 4839 cells from the somatosensory cortex, hippocampus, and ventricle from a CD-1 mouse at age of p22 was conducted [37]. Four thousand three hundred eighty-eight cells from somatosensory cortex were extracted with 33 genes detected where all of the FISH genes were overlapped with the 13,997 genes, including *GAD2*, *SLC32A1*, *CRHBP*, *CNR1*, *VIP*, *CPNE5*, *PTHLH*, *CRH*, *TBR1*, *LAMP5*, *RORB*, *SYT6*, *KCNIP2*, *ALDOC*, *GFAP*, *SERPINF1*, *MFGE8*, *SOX10*, *PLP1*, *PDGFRA*, *BMP4*, *ITPR2*, *TMEM2*, *CTPS*, *ANLN*, *MRC1*, *HEXB*, *TTR*, *FOXJ1*, *VTN*, *FLT1*, *APLN*, and *ACTA2*. RNA-seq data can be extracted from http://loom.linnarssonlab.org/clone/Mousebrain.org.level1/L1_Cortex2.loom. The FISH data can be found at http://linnarssonlab.org/osmFISH/availability/.

#### PBMC (2638 freeze-thaw human PBMC cells by 10X Genomics)

Two thousand seven hundred freeze-thaw peripheral blood mononuclear cells (PBMC) from a healthy donor were profiled by 10X, where 32,738 genes were detect [38]. Cells expressed less than 200 or greater than 2500 genes or have > 5% mitochondrial counts were removed (2638 cells left) and 8654 genes were left after gene selection. RNA-seq data can be found at https://support.10xgenomics.com/single-cell-gene-expression/datasets/1.1.0/frozen_pbmc_donor_a.

#### CBMC (GSE100866, 8005 human CBMC cells by CITE-seq)

Cord blood mononuclear cells were profiled by CITE-seq, where 8005 human cells were detected in total. We used all detected human (20,400) genes (11,556 genes were left after gene selection) for down-sampling [39]. RNA-seq data can be found at GSE100866.

#### JURKAT_293T (3258 and 2885 human cells by 10X Genomics)

Jurkat and 293T were profiled by 10X separately. Cells expressed less than 500 genes were removed and 3258 Jurkat and 2885 293 T cells were left [15]. After gene selection, Jurkat, 293T, and their merged data left 11,293, 11,974, and 13,328 genes, respectively. Single-cell RNA-seq data can be found at https://support.10xgenomics.com/single-cell-gene-expression/datasets/1.1.0/jurkat and https://support.10xgenomics.com/single-cell-gene-expression/datasets/1.1.0/293t while the corresponding bulk RNA-seq data can be found at GSE129240.

#### 10X_5CL (GSE126906, 3918 cells from 5 cell lines by 10X Genomics)

Five thousand one cells from 5 human lung adenocarcinoma cell lines H2228, H1975, A549, H838, and HCC827 were profiled by 10X, where 32,895 genes were detected. Cells expressed less than 500 genes were removed (3918 cells left) and 18,296 genes were left after gene selection [15]. Single-cell RNA-seq data can be found at GSE126906 and the corresponding bulk RNA-seq data can be found at GSE86337.

#### BONE_MARROW (HCA, 6939 human bone marrow cells by 10X Genomics)

Six thousand nine hundred forty-one human bone marrow cells from sample MantonBM6 were profiled by 10X [15, 40], where 32,738 genes were detected. Cells expressed less than 500 genes were removed (6939 cells left) and 13,813 genes were left after gene selection. The original single-cell RNA sequencing data provided by HCA was aligned to hg19 and the expression matrix after cell filtering can be found at https://drive.google.com/file/d/1euh8YB8ThSLHJNQMTCuuKp_nRiME1KzN/view?usp=drive_web. The corresponding bulk RNA-seq used for cell identification can be found at GSE74246.

#### RERINA (GSE63473, 49,300 retina STAMPs by Drop-seq)

Retinas of mice at age of p14 were profiled in 7 different replicates by Drop-seq, where 6600, 9000, 6120, 7650, 7650, 8280, and 4000 STAMPs (single-cell transcriptomes attached to micro-particles) were collected with 24,658 genes detected [2]. Cells were merged and 14,871 genes were left after gene selection. 44,808 cells labeled STAMPs were used for evaluation. RNA-seq data can be found at GSE63473.

#### BRAIN_SPLiT (GSE110823, 156,049 mouse brain and spinal cord nuclei by SPLiT-seq)

156,049 mice nuclei from developing brain and spinal cord at age of p2 or p11 mice were profiled by SPLiT-seq, where 26,894 genes were detected [3], in which 15,025 genes were left after gene selection. RNA-seq data can be found at GSE110823.

#### BRAIN_1.3 M (1,282,594 mouse brain cells by 10X Genomics)

1,306,127 cells from combined cortex, hippocampus, and subventricular zone of 2 E18 C57BL/6 mice were profiled by 10X, where 27,998 genes were detected [38]. Cells expressed less than 500 or greater than 5000 genes were removed and 15,080 genes were left after gene selection, and 1,282,594 cells were kept for further analysis. RNA-seq data can be found at https://support.10xgenomics.com/single-cell-gene-expression/datasets/1.3.0/1M_neurons.

#### DISC

DISC is implemented in Python and builds on Google TensorFlow. It runs on both CPUs and GPUs. The source code and the datasets are available at https://github.com/xie-lab/DISC [41] and Zenodo [42] under Apache License 2.0.

## Supplementary information

**Additional file 1.** Supplementary figures and tables for DISC architecture and computational experiments.

**Additional file 2.** Review history.
