# Supplementary material for: DISC: a highly scalable and accurate inference of gene expression and structure for single-cell transcriptomes using semi-supervised deep learning
Source: Genome Biol. 2020 Jul 10;21:170. doi: 10.1186/s13059-020-02083-3 (PMC7353747; doi:10.1186/s13059-020-02083-3)
Supplement: Supplementary file 1 — Additional file 1. Supplementary figures and tables for DISC architecture and computational experiments. [file 13059_2020_2083_MOESM1_ESM.docx]

**Supplementary Information**

**DISC: a highly scalable and accurate inference of gene expression and structure for single-cell transcriptomes using semi-supervised deep learning**

**
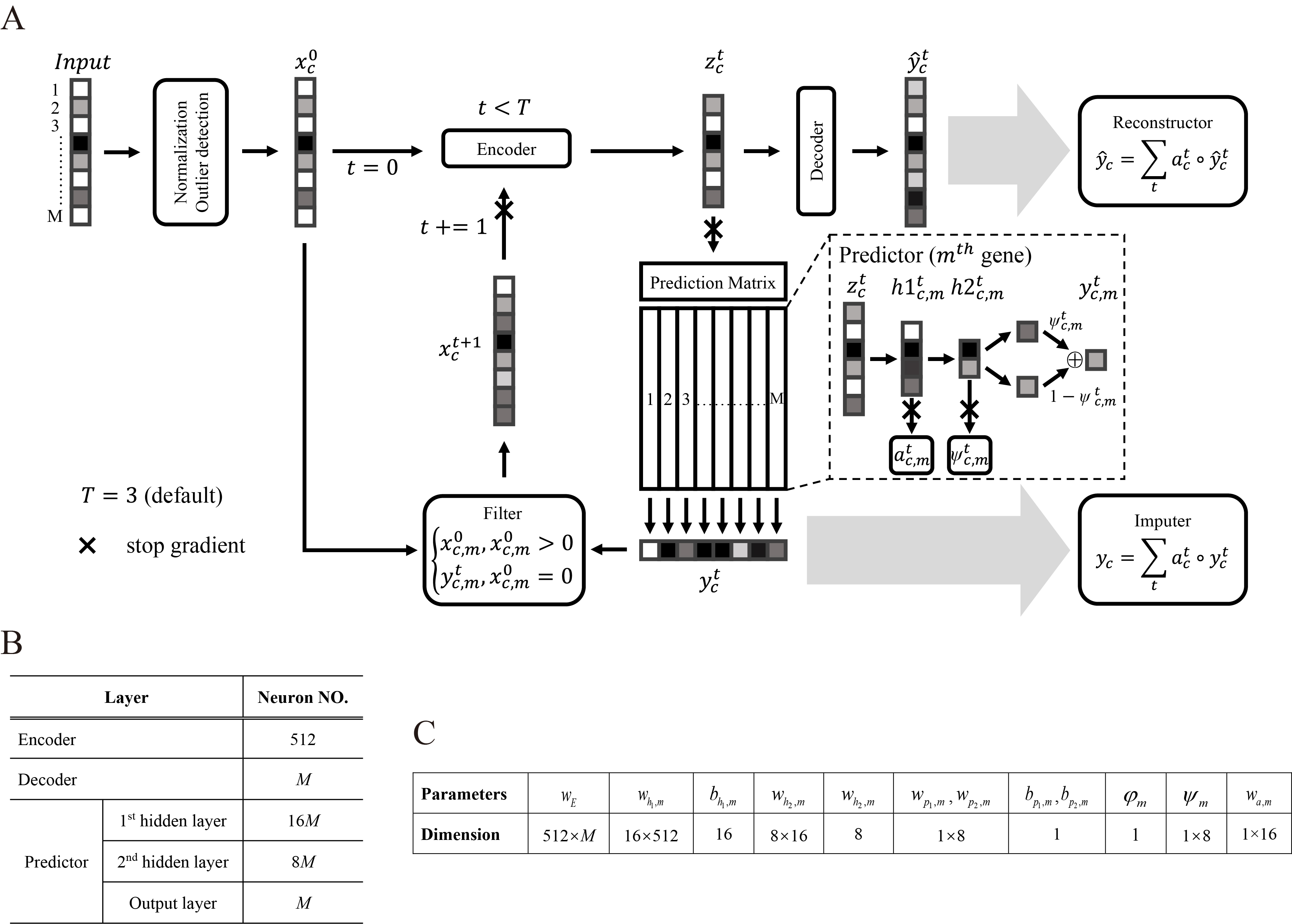
**

**Fig. S1** (A) Model structure of DISC. (B) DISC architecture parameters. (C) Dimension of learnable parameters


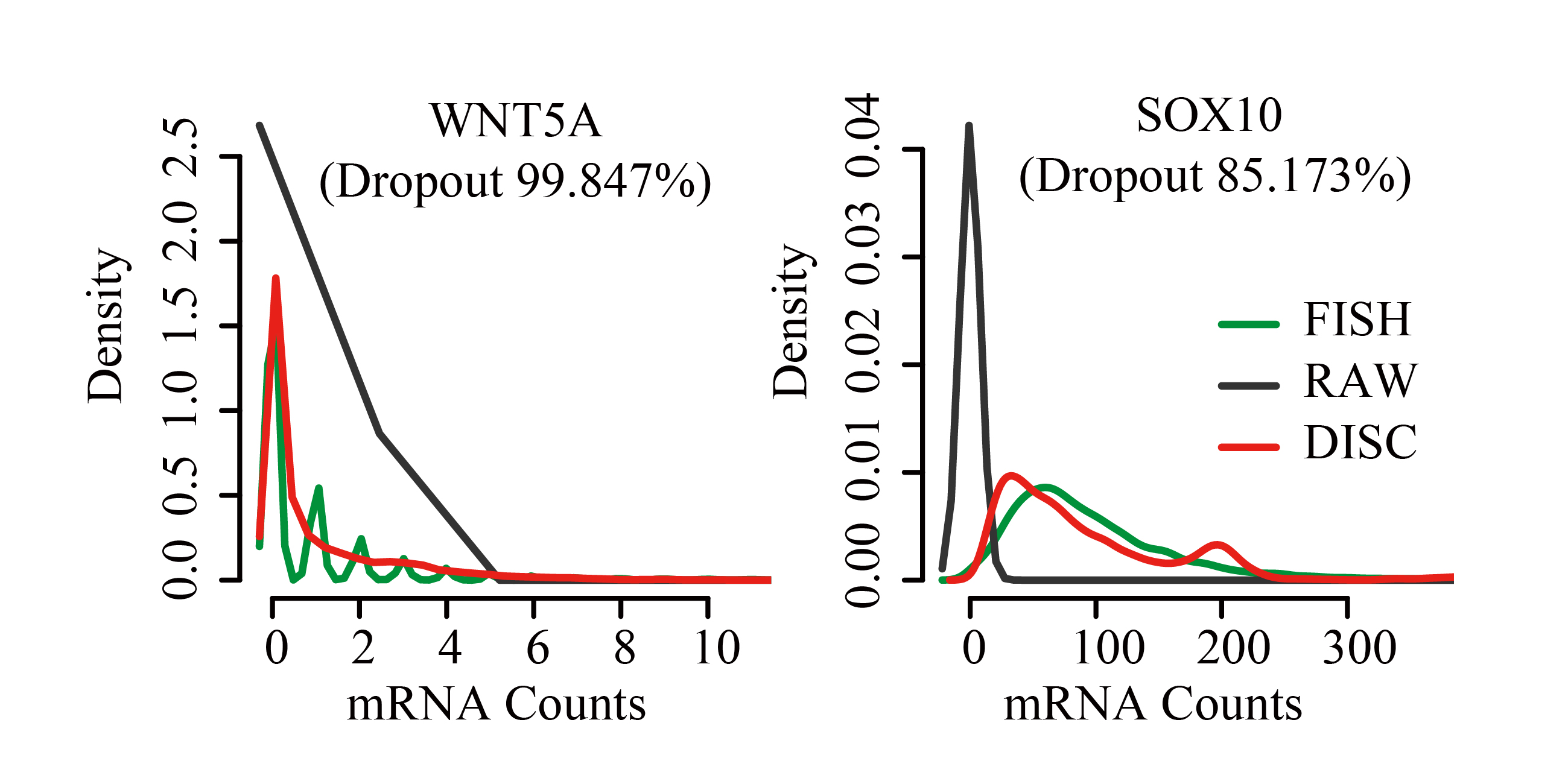


**Fig. S2** Gene distribution of WNT5A (left) and SOX10 (right) genes among FISH, the raw scRNA-seq (RAW) and DISC. The dropout levels of two genes are shown in the title.


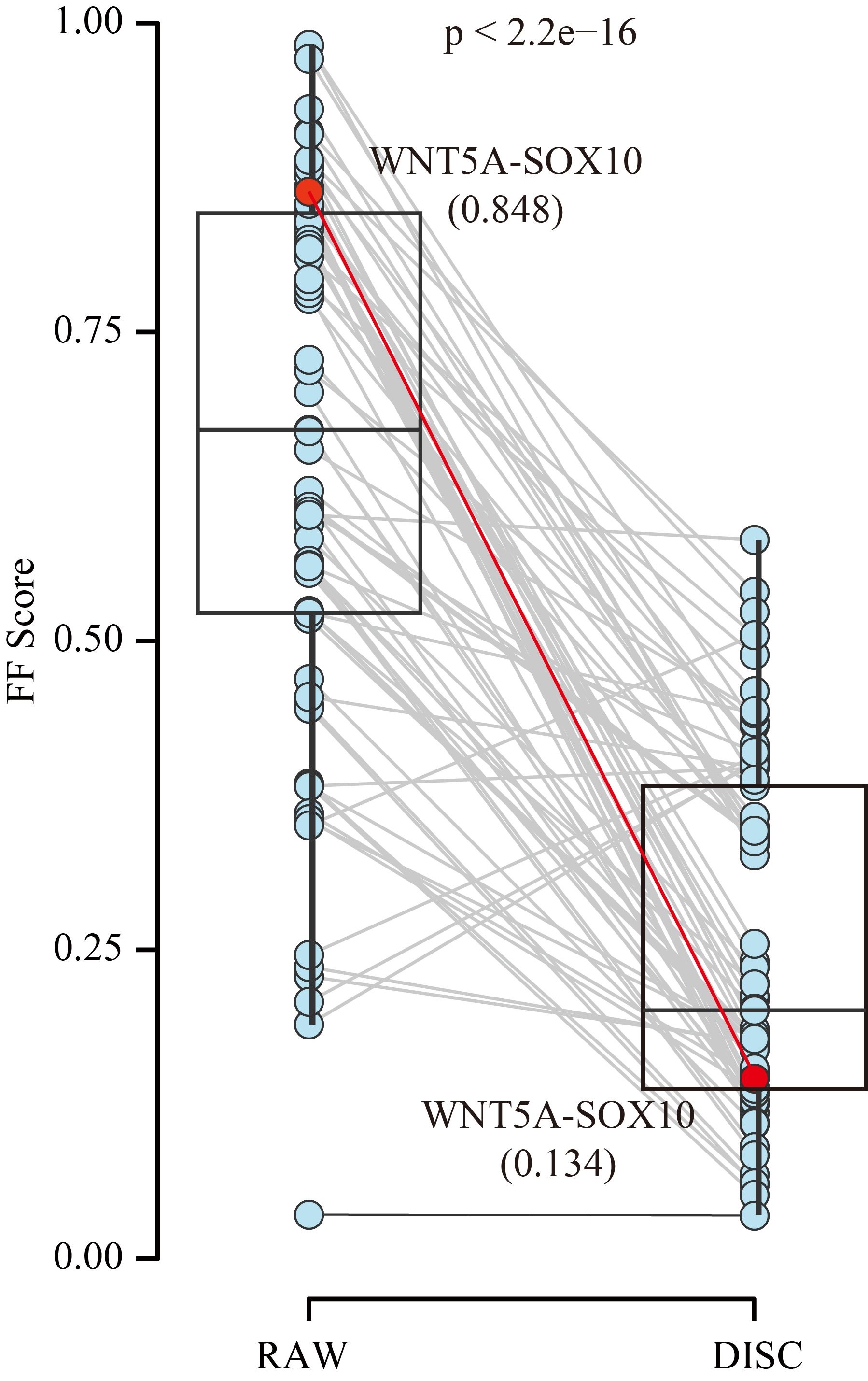


**Fig. S3** FF score distribution for 81 gene-pairs between the RAW and DISC against FISH.


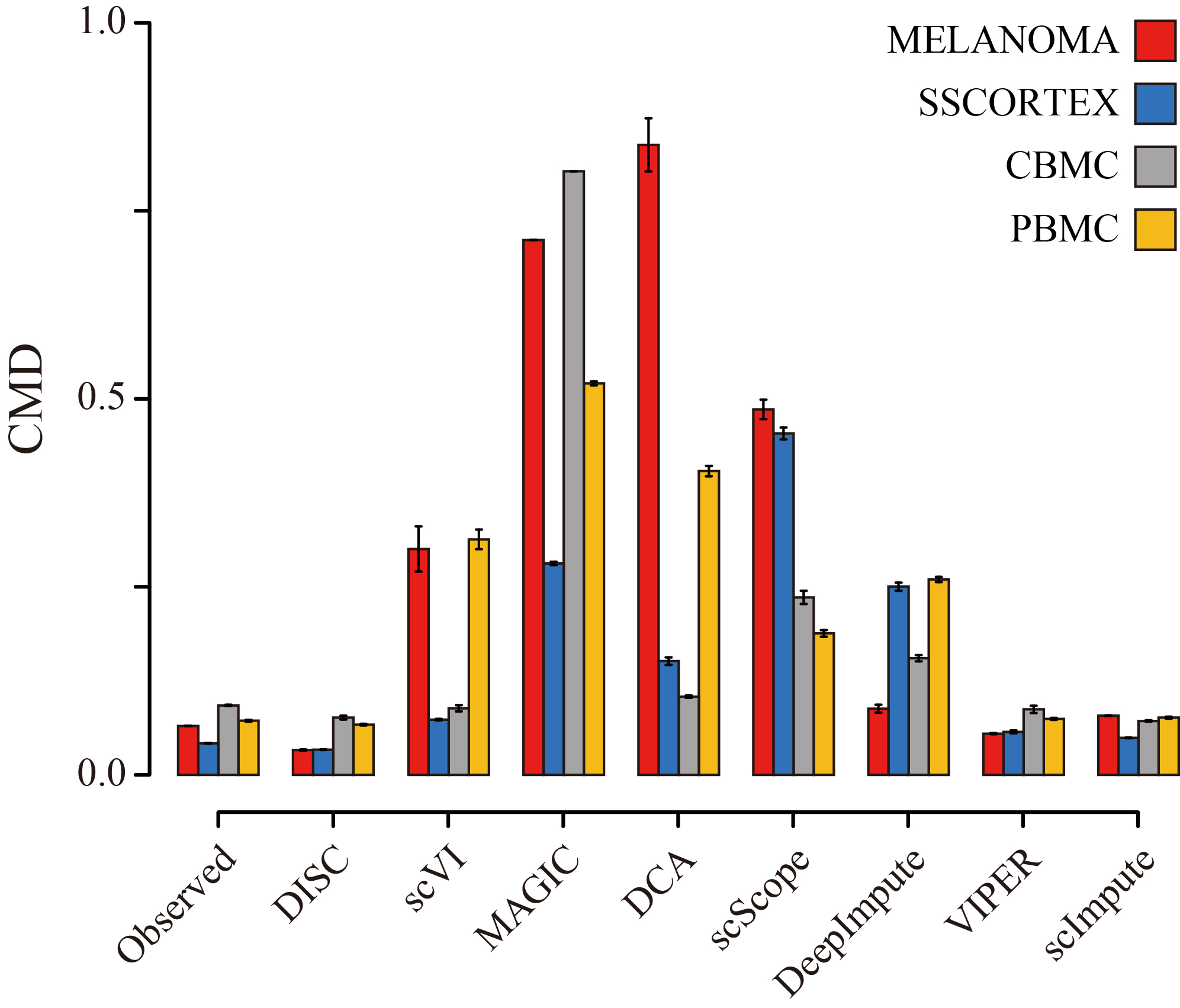


**Fig. S4** CMD of gene co-expression between the reference and the observed/the imputation.


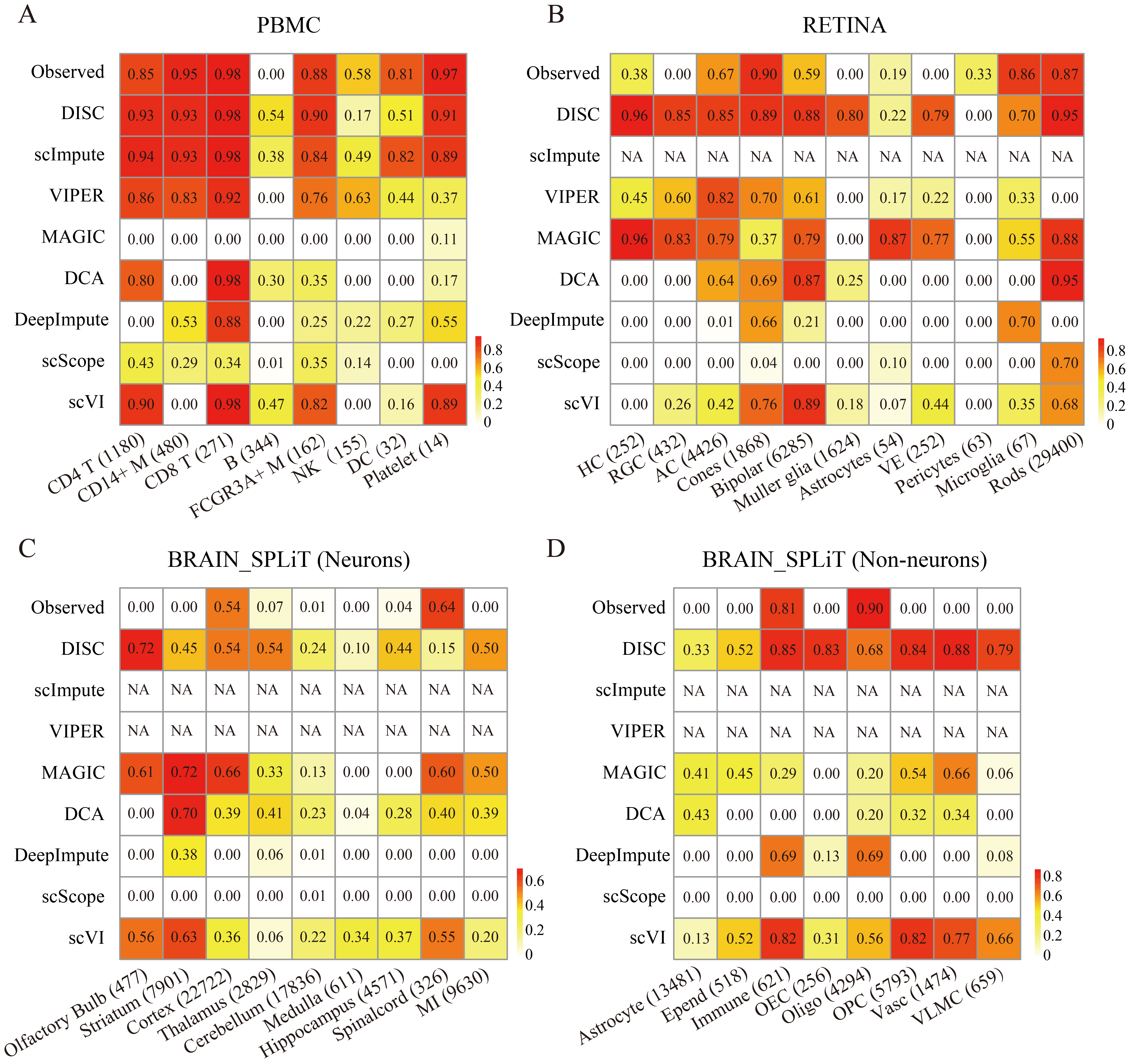


**Fig. S5** Jaccard index of each cell type for down-sampling to 30% of the reference. Names and numbers of cell types are given in the label of x-axis. NA: failed to impute. (**A**) PBMC dataset; (**B**) RETINA dataset. (**C**) neurons of BRAIN_SPLiT and (**D**) non-neurons of BRAIN_SPLiT.

**
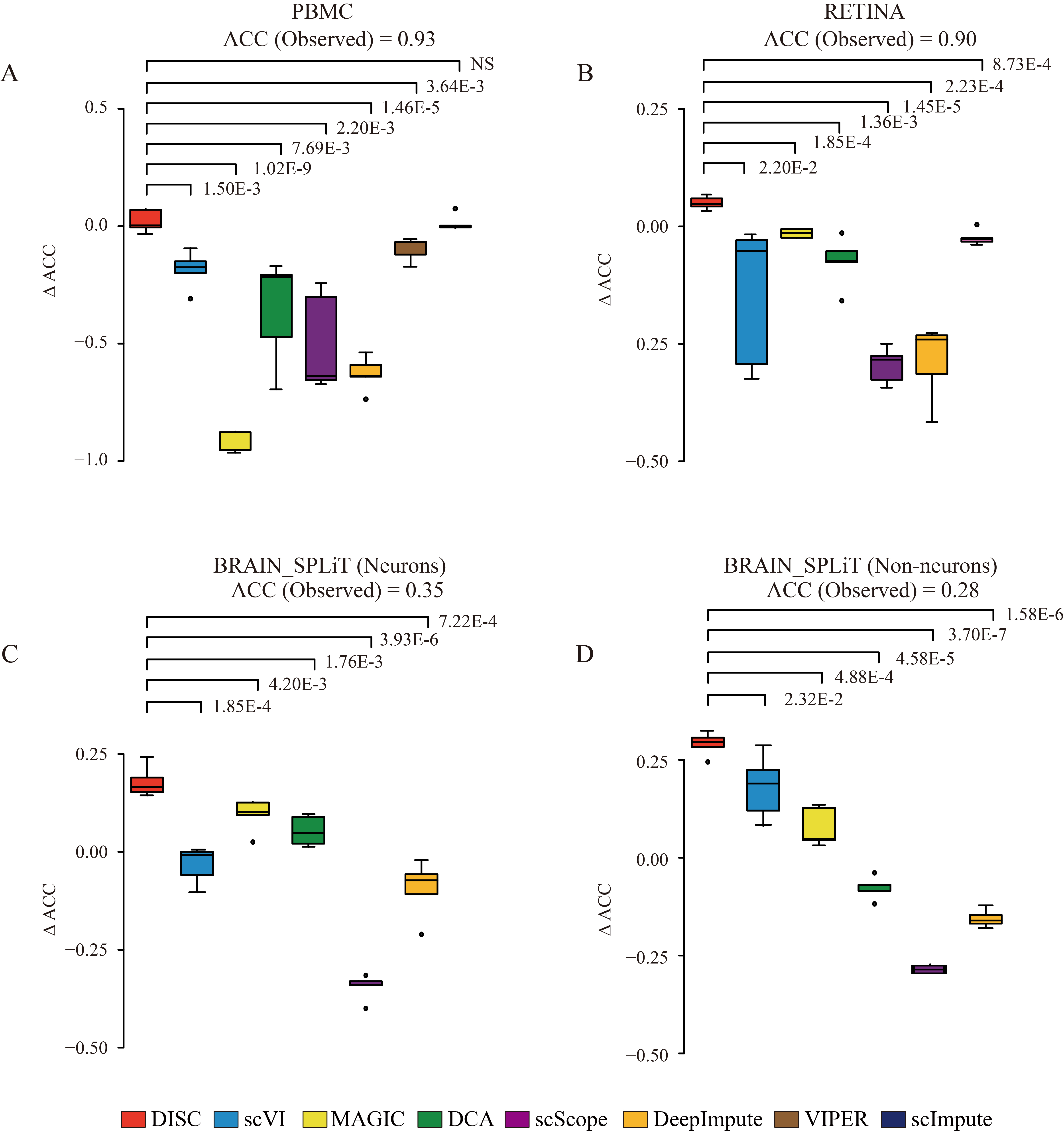
**

**Fig. S6** Evaluation of cell type identification after down-sampling to 50% of the reference. ACC was used to measure accuracy of cell type identification. Y-axis showed the difference of ACC between the imputed and the observed datasets. ACCs of each observed dataset are shown. P values were calculated using one tailed paired t test. (**A**) PBMC dataset; (**B**) RETINA dataset. (**C**) neurons of BRAIN_SPLiT and (**D**) non-neurons of BRAIN_SPLiT. The following approaches failed due to ‘out-of-memory” error: scImpute on RETINA, VIPER and scImpute on BRAIN_SPLiT.

**
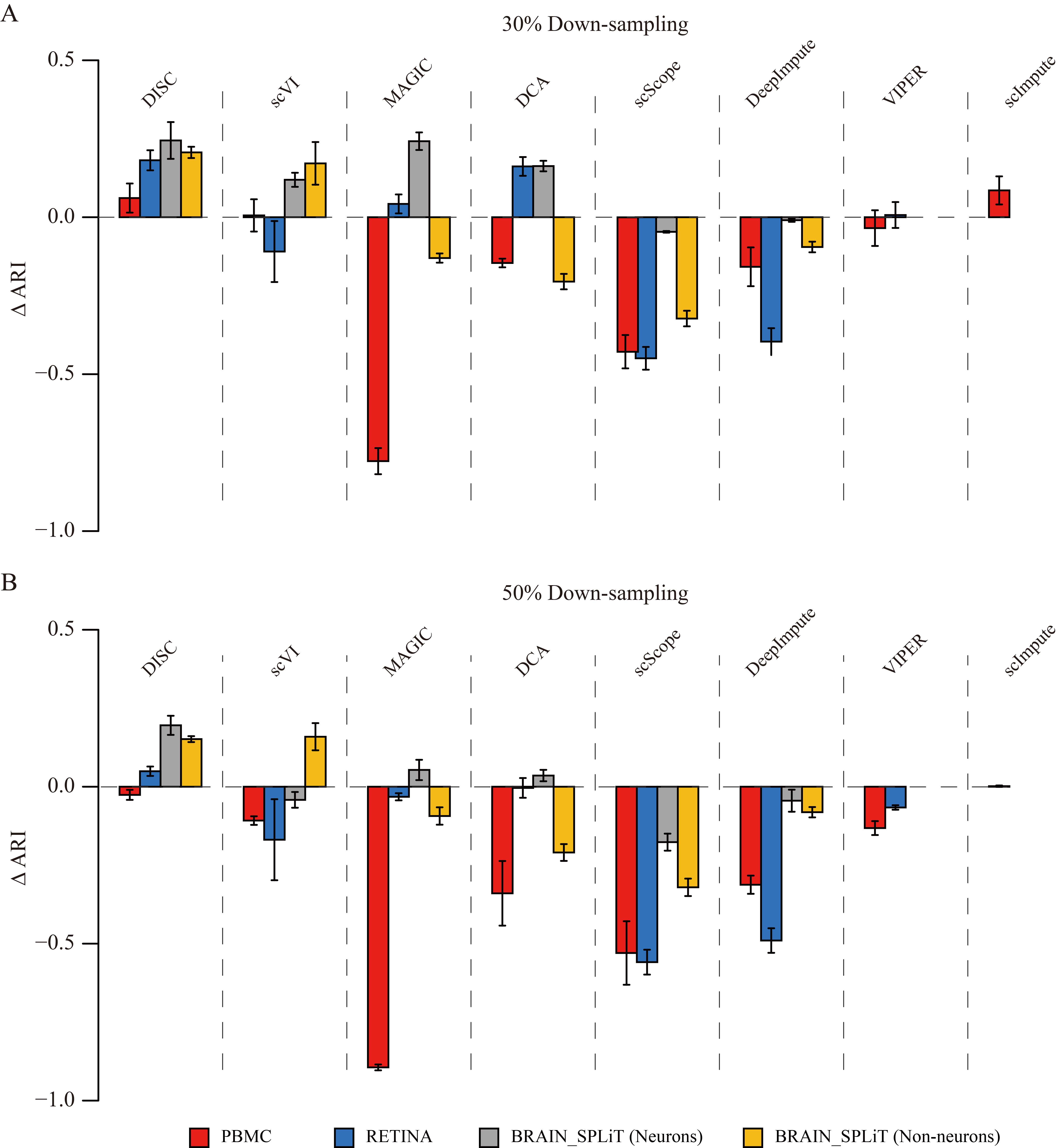
**

**Fig. S7** Evaluation of cell type identification by ARI after down-sampling to 30% and 50%. Y-axis showed the difference of ARI between the imputed and the observed datasets. (**A**) Down-sampling to 30% of the reference, where the ARI of the observed are 0.81, 0.72, 0.05 and 0.31 for the PBMC, RETINA, Neurons and Non-neurons datasets, respectively. (**B**) Down-sampling to 50% of the reference, where the ARI of the observed are 0.92, 0.85, 0.18 and 0.30 for the PBMC, RETINA, Neurons and Non-neurons datasets, respectively.


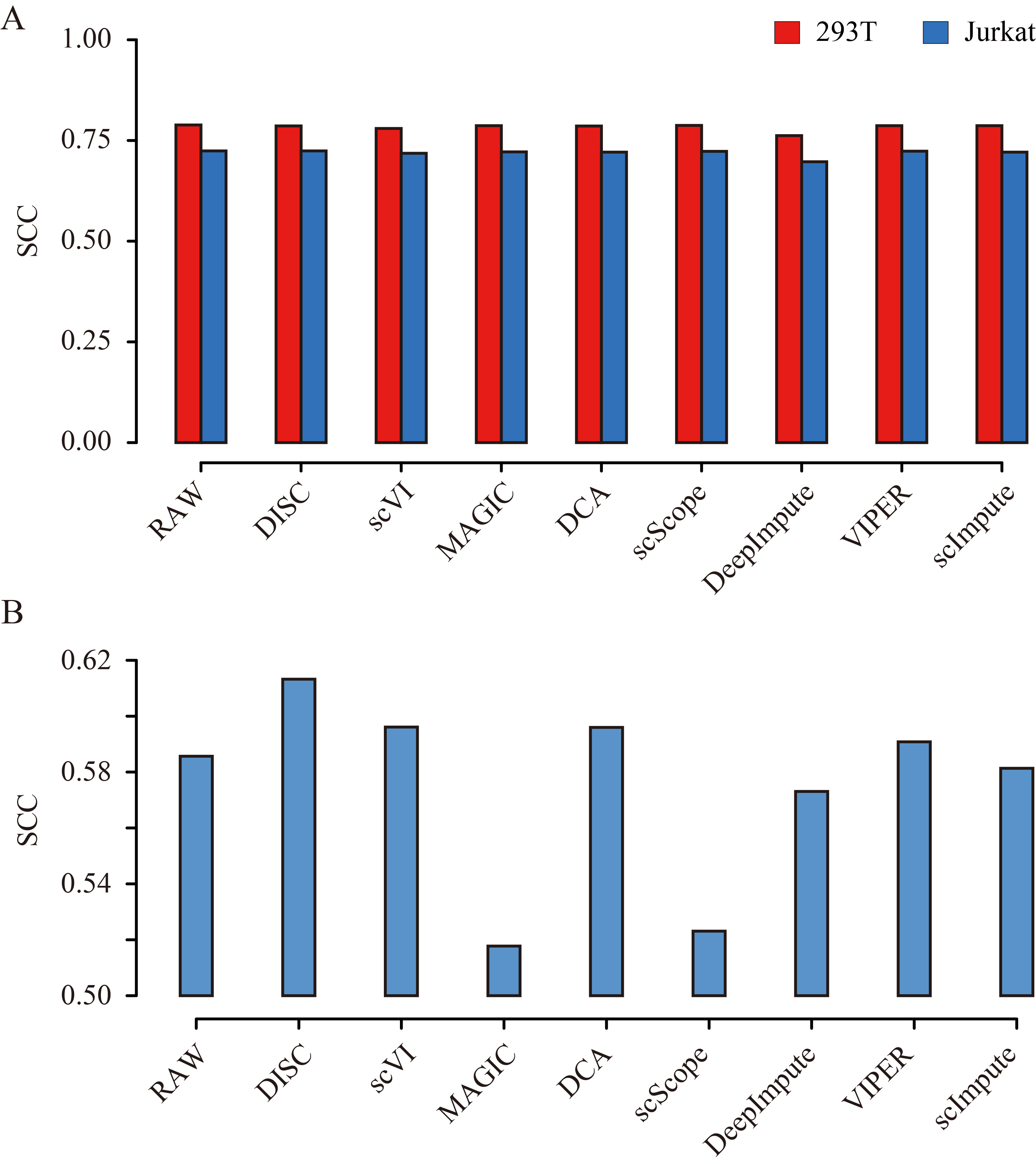


**Fig. S8** Spearman correlation coefﬁcient (SCC) between the imputed scRNA-seq profiles and the bulk RNA-seq profiles (**A**) for Jurkat and 293T cell lines and (**B**) for expression difference between the Jurkat and 293T cell lines.


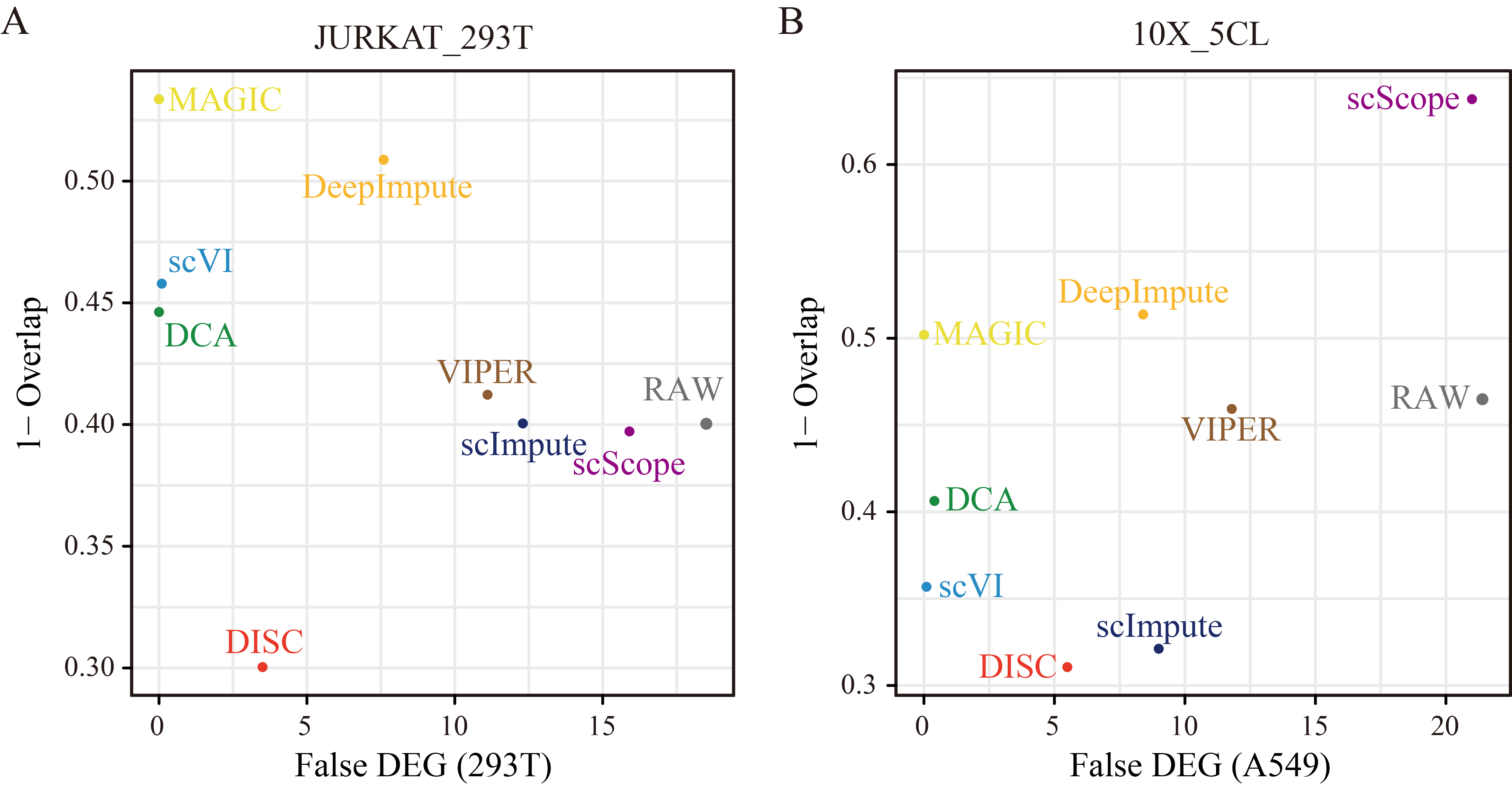


**Fig. S9** DEG identified using Wilcoxon method for (A) the JURKAT_293T dataset and (B) the 10X_5CL dataset, where x-axis shows the false number of DEGs identified from a homogeneous population (293T and A549 cell lines were used, respectively) and y-axis shows the averaged overlaps of DEGs identified by scRNA-seq data to that of bulk RNA-seq for all the combination of cell lines in the dataset.


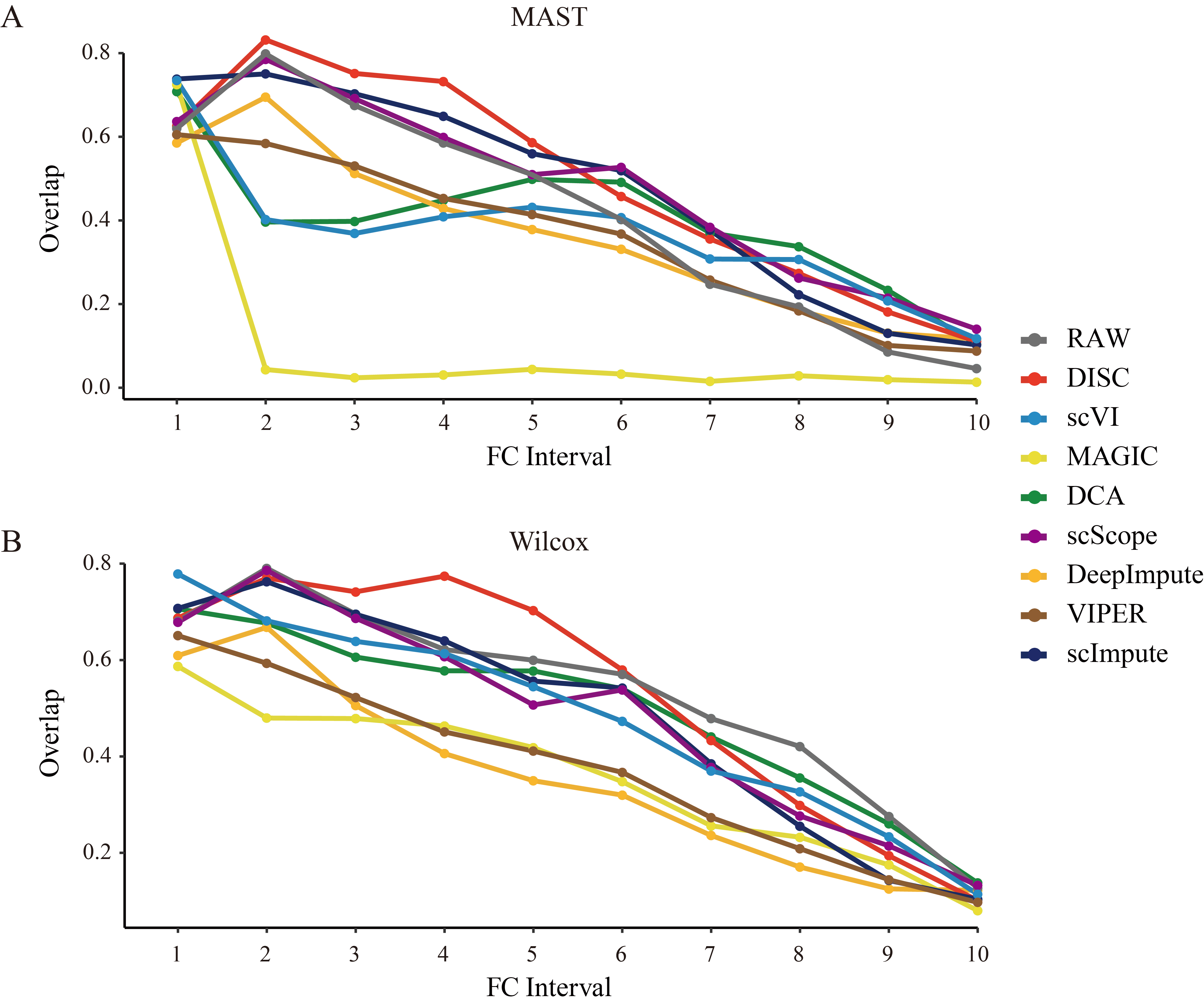


**Fig. S10** For a pair of cell lines in the 10X_5CL dataset, genes were grouped into 10 intervals ranked by the FC values. The overlap between bulk and single-cell DEGs identified using (**A**) MAST and (**B**) Wilcoxon methods were calculated for 10 combinations of 5 cell lines in the 10X_5CL dataset for each interval.

**
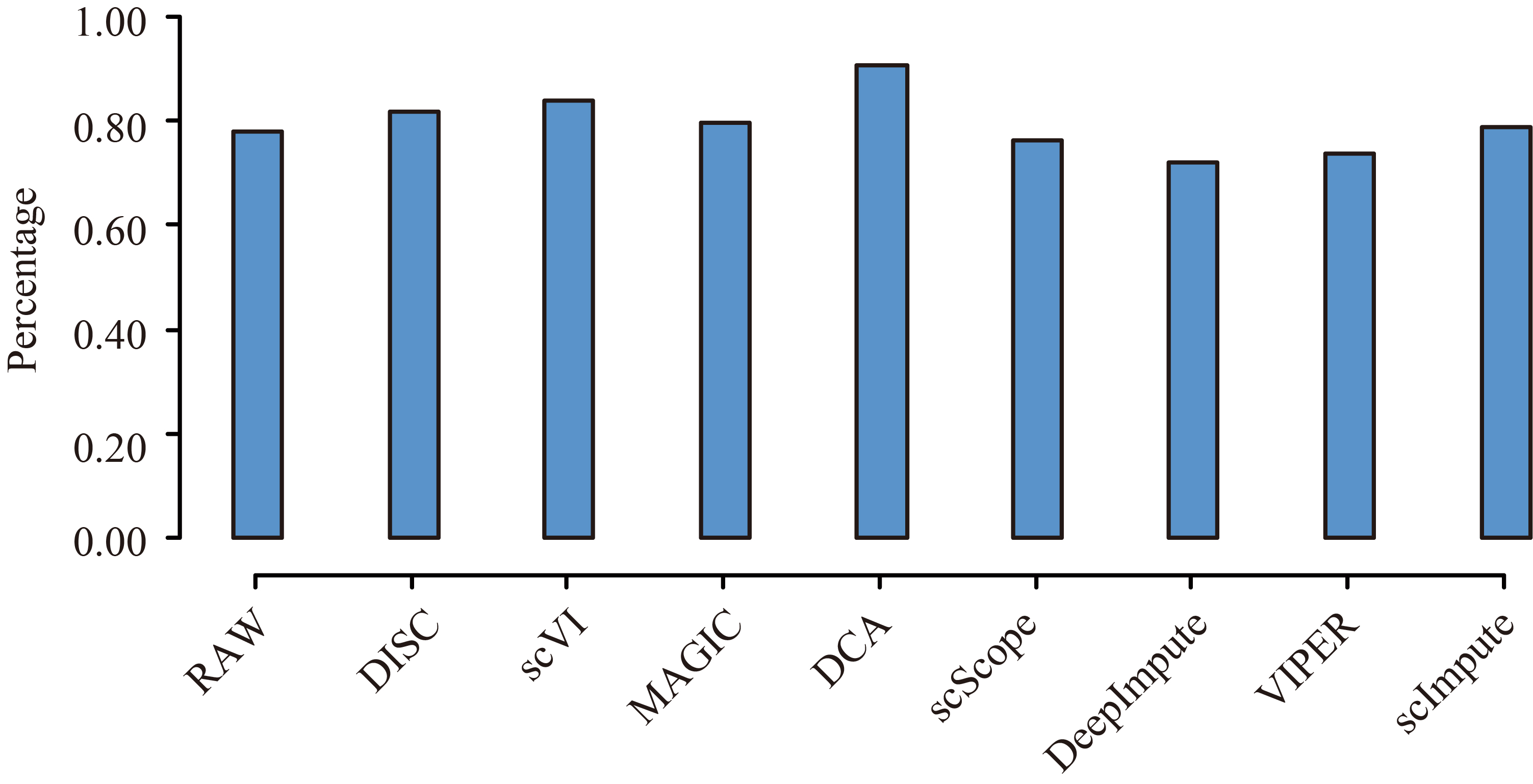
**

**Fig. S11** Inference of the pseudo-temporal order for cells in the BONE_MARROW dataset by Monocle2 and comparison with the known cell differentiation order. The known differentiation levels (HSC: level 1; MPP: level 2; LMPP and CMP: level 3; CLP, GMP, and MEP: level 4; B cell, CD4 T cell, CD8 T cell, NK cell, Monocyte, and Erythroid: level 5) served as the reference. Y-axis shows the percentage of correctly ordered cell-pairs.

**
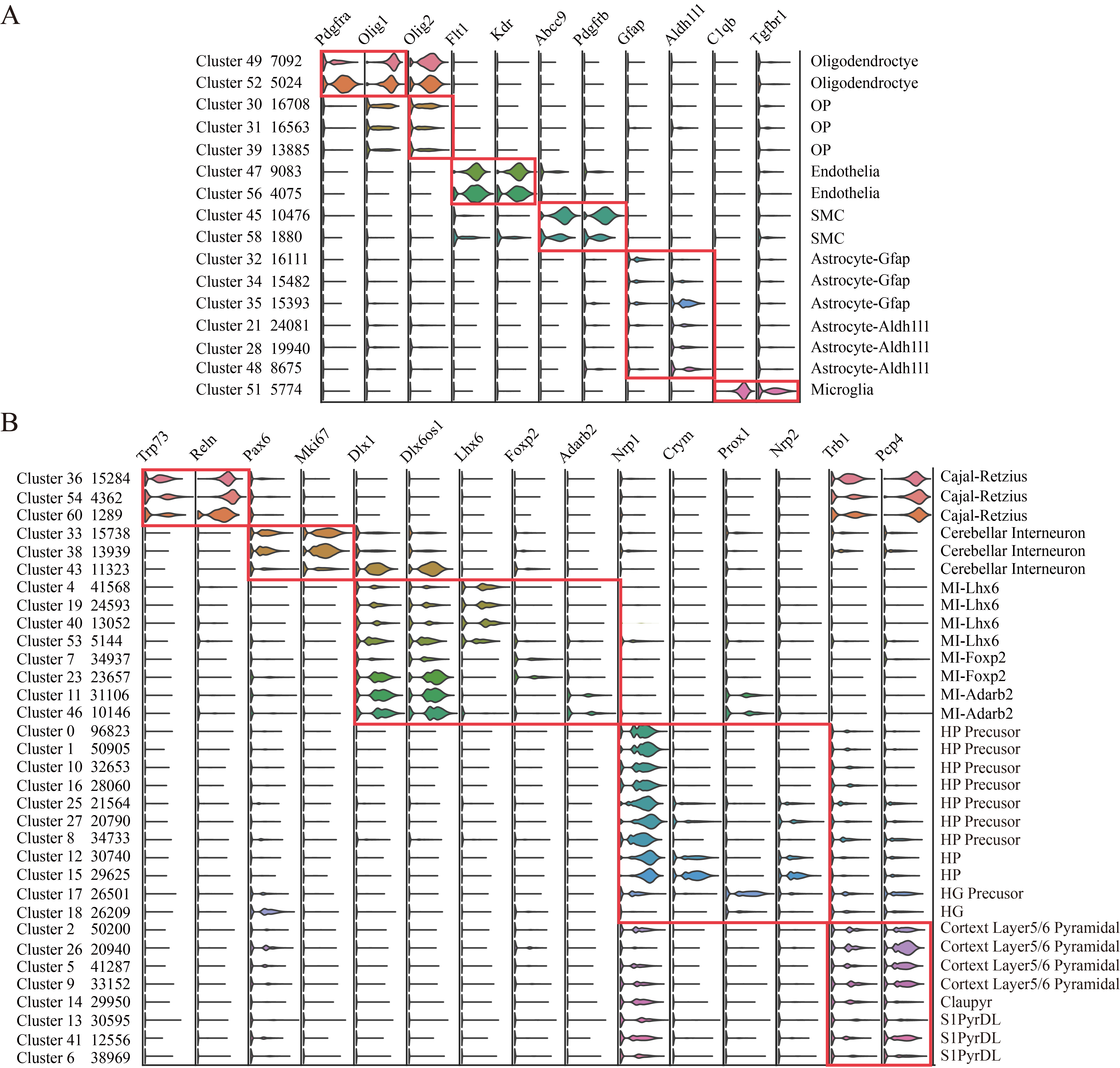
**

**Fig. S12** Expression distribution of marker genes in BRAIN_1.3M dataset. The cluster ID and the number of cells in each cluster are shown on the left; the annotated cell type for each cluster is shown on the right. Red box represents the same cell type. (**A**) clusters for the non-neuronal cells. (**B**) clusters for the neuronal cells.

**
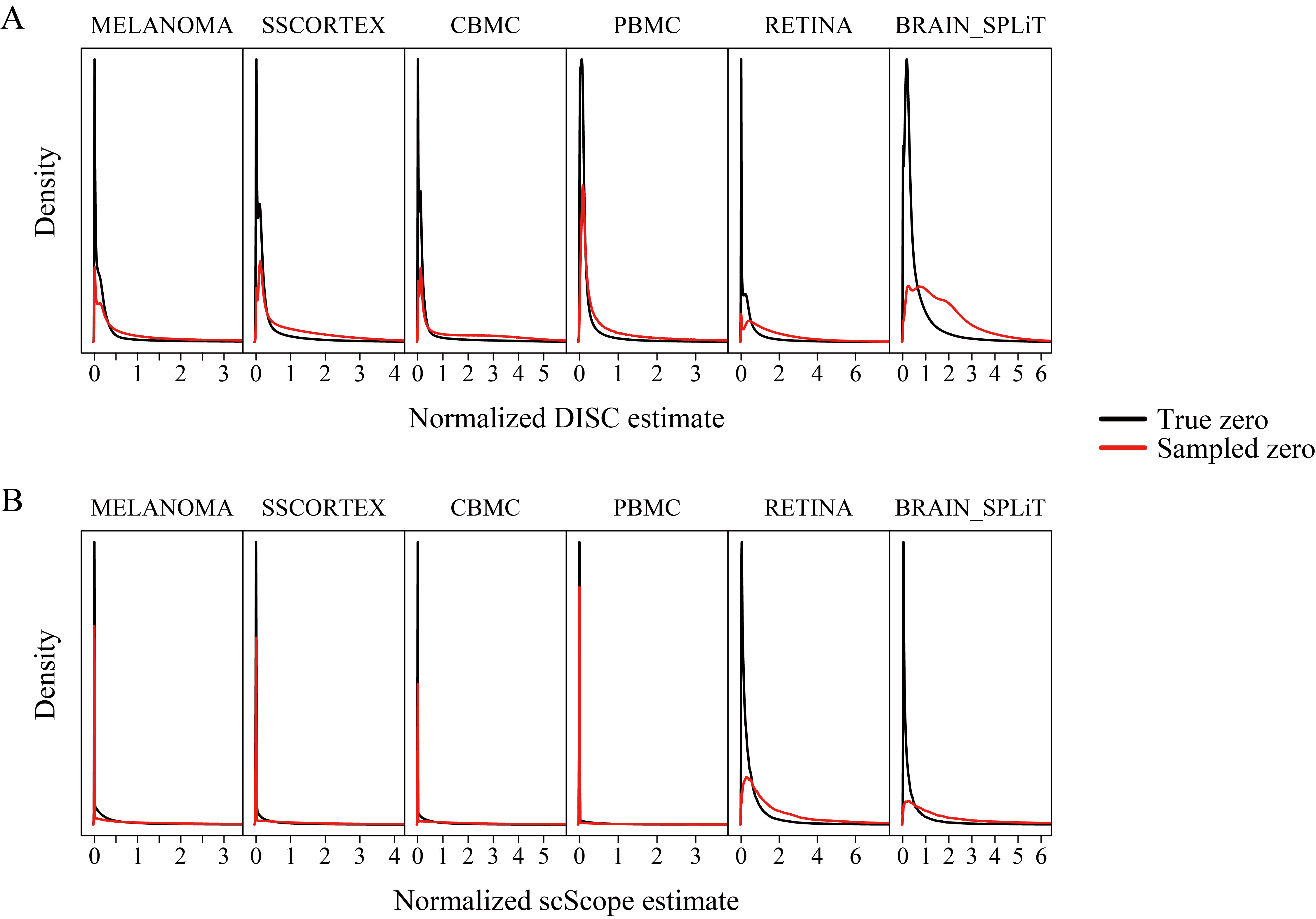
**

**Fig. S13** Density plots of imputed gene expression of true zeros (zero-count genes in RAW) and sampled zeros (positive-count genes in RAW becomes zero-count genes after sampling in down-sampled data) for all the genes in 6 datasets. (**A**) DISC and (**B**) scScope.

**
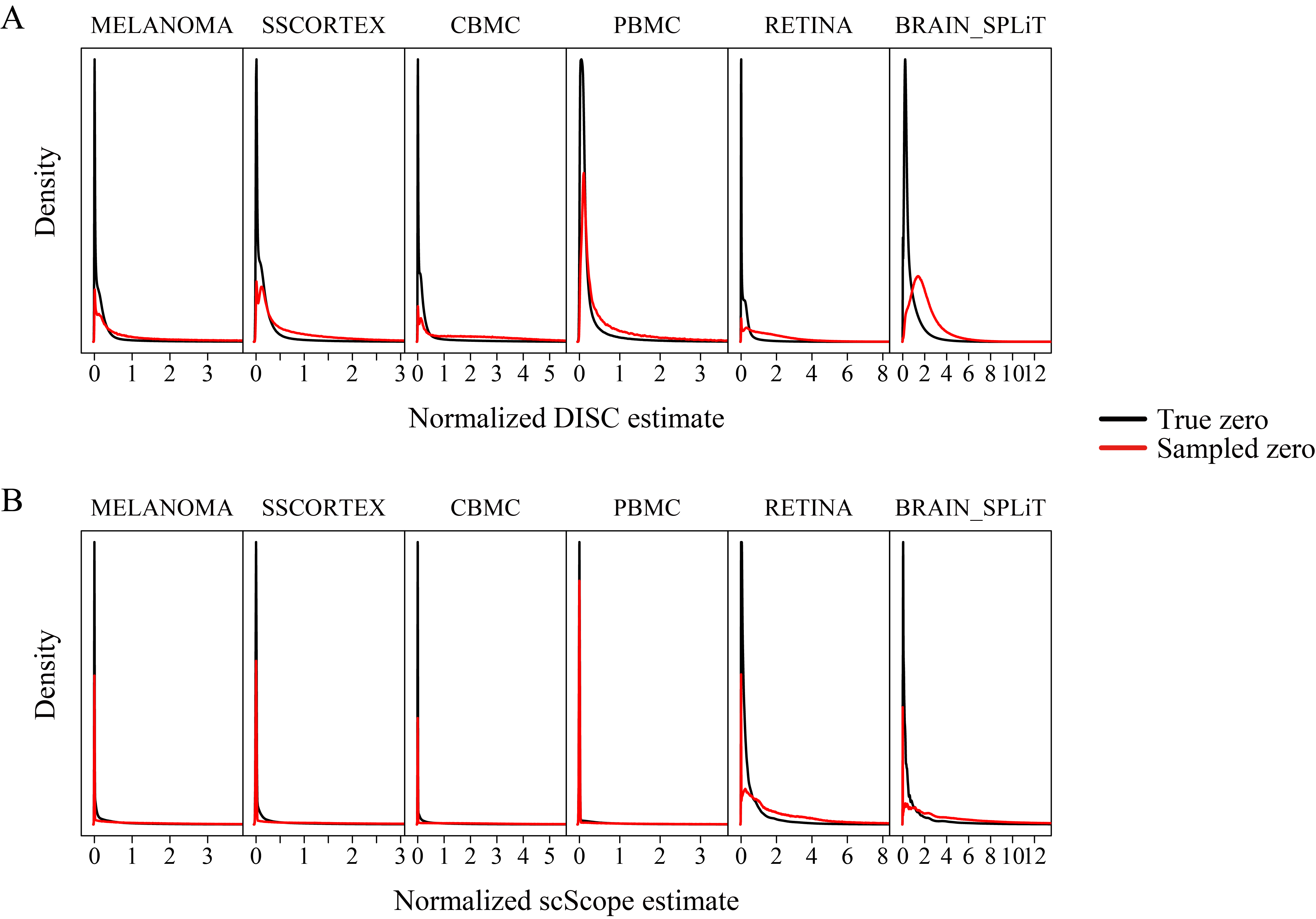
**

**Fig. S14** Density plots of imputed gene expression of true zeros (zero-count genes in RAW) and sampled zeros (positive-count genes in RAW becomes zero-count genes after sampling in down-sampled data) for the top 1000 highly variable genes in 6 datasets. (**A**) DISC and (**B**) scScope.

**Table S1：**Mean library size of raw and down-sampling of the datasets.

| **Datasets** | | **MELANOMA** | **SSCORTEX** | **CBMC** | **PBMC** | **RETINA** | **BRAIN_SPLiT** |
| --- | --- | --- | --- | --- | --- | --- | --- |
| Platforms | | Drop-seq | 10X | Cite-seq | 10X | Drop-seq | SPLiT-seq |
| Library Size (Mean) | Raw | 2213.30 | 3444.34 | 2327.77 | 2372.58 | 1287.41 | 1329.02 |
|  | DS 0.5 | 1106.64 | 1722.18 | 1163.88 | 1186.30 | 643.71 | 664.51 |
|  | DS 0.3 |  |  |  | 711.77 | 386.22 | 398.71 |

DS 0.5: down-sampling to 50% of the reference; DS 0.3: down-sampling to 30% of the reference.

**Table S2：**Gene markers used to annotate cell types for the RETINA dataset.

| **Cell Type** | **Marker Genes** | **Cells** | **Reference** |
| --- | --- | --- | --- |
| Horizontal cells | *Lhx1* & *Pax6* | 252 | [1, 2] |
| Retinal ganglion cells | *Slc17A6* & *Pax6* | 432 |  |
| Amacrine cells | (*Pax6* & *Gad1*) *∩* (*Pax6* & *Slc6a9*) | 4,426 |  |
| Cones | *Opn1mw* | 1,868 |  |
| Bipolar cells | *Vsx2* | 6,285 |  |
| Muller glia | *Pax6* & *Vsx2* & *Rlbp1* | 1,624 |  |
| Astrocytes | *Rlbp1* & *Gfap* | 54 |  |
| Fibroblasts | *Pax6* & *Slc6a9* & *Vsx2* | 85 |  |
| Vascular endothelium | *Pecam1* & *Kcnj8* | 252 |  |
| Pericytes | *Kcnj8* | 63 |  |
| Microglia | *Cx3cr1* | 67 |  |
| Rods | no these marker genes | 29,400 |  |

**Table S3：**Gene markers used to annotate cell types for the PBMC dataset.

| **Cell Type** | **Marker Genes** | **Cells** | **Reference** |
| --- | --- | --- | --- |
| CD4 T | *IL7R* & ^*CD8A* | 1,180 | https://satijalab.org/seurat/v3.1/pbmc3k_tutorial.html |
| CD14+ Mono | *CD14*& *LYZ* | 480 |  |
| B | *MS4A1* | 344 |  |
| CD8 T | *CD8A* | 271 |  |
| FCGR3A+ Mono | *FCGR3A* & *MS4A7* | 162 |  |
| NK | *GNLY* & *NKG7* & ^*CD8A* | 155 |  |
| DC | *FCER1A* & *CST3* | 344 |  |
| Platelet | *PPBP* | 271 |  |

**Table S4：**Gene markers used to annotate cell types for the BRAIN_SPLiT dataset.

| **Class** | **Cell Type** | **Marker Genes** | **Cells** | **Reference** |
| --- | --- | --- | --- | --- |
| Neuron | Olfactory bulb | *Deptor* | 477 | [3] |
|  | Striatum | *Rarb* | 7,901 |  |
|  | Cartex | *Satb2* | 22,722 |  |
|  | Rostral Midbrain | *Tfap2d* | 736 |  |
|  | Thalamus | *Fign* | 2,829 |  |
|  | Cerebellum | *Arap1 \|\| Pax3*  \|\| *Ntn1* | 17,836 |  |
|  | Medulia | *Pax2 & ^Ntn1* | 611 |  |
|  | Basal Ganglia | *Ntn1* & *Slc6a3* | 47 |  |
|  | Hippocampus | *Fn1* \|\| *Tspan18* | 4,571 |  |
|  | SpinalCord | *Pdella* | 326 |  |
|  | Mirgrating Interneuron | *Dlx6os1* | 9,630 |  |
| Non-neuron | Oligo | *Mbp* | 4,294 |  |
|  | OPC | *Pdgfra* | 5,793 |  |
|  | Immune | *Dock2* | 621 |  |
|  | VLMC | *Rgs5* | 256 |  |
|  | Vasc. | *Col1a2* | 1,474 |  |
|  | Astrocyte | *Aldh1l1* | 13,481 |  |
|  | Epend. | *Dnah11* | 518 |  |
|  | OEC | *Mybpc1* | 256 |  |

**Table S5：**Number of clusters and cells included in three major cell groups of BRAIN_1.3M dataset. Gene markers and criteria from the Allen Brain Atlas (http://brainmap.org) were used to annotate three major cell groups [1].

| **Cell Type** | **Clusters** | **Cells** | **%** | **Criteria** |
| --- | --- | --- | --- | --- |
| Glutamatergic neurons | 0,1,2,3,5,6,8,9,10,12,13,14,15,16,17,18,22,24,25,26,27,29,36,37,41,42,54,60 | 816,062 | 63.63% | max(*Slc17a6*, *Slc17a7*) > max(*Gad1*, *Gad2*, *Olig1*, *Gja1*, *Xdh*, *Ctss*, *Myl9*, *Slc32a1*) |
| GABAergic neurons | 4,7,11,19,23,33,  38,40,43,46,53,57 | 229,148 | 17.87% | max(*Gad1*, *Gad2*, *Slc32a1*) > max(*Slc17a7*, *Slc17a6*, *Olig1*, *Gja1, Xdh*, *Ctss*, *Myl9*) |
| Non-neurons | 20,21,28,30,31,32,34,35,39,44,45,47,48,49,50,51,52,55,56,58,59 | 237,384 | 18.51% | Other cell types |

**Table S6：**Identification of cell types based on the clusters discovered by DISC on the BRAIN_1.3M dataset.

| **Clusters** | **Class** | **Cell Type** | **Gene Markers** | **Reference** |
| --- | --- | --- | --- | --- |
| 36,54,60 | Neuron | Cajal-Retzius | *Trp73* & *Reln* | [4] |
| 33,38,43 |  | Cerebellar Interneuron Progenitor | *Mki67* | [5] |
| 4,19,40,53,7,23,11,46 |  | Migrating Interneuron | (*Dlx1,2,5,6,6os1*) & *Lhx6* & *Foxp2* & *Adarb2* | [6] |
| 0,1,8,10,16,25,27 |  | Hippocampal Pyramidal Precursor | *Nrp1* | [7, 8] |
| 12,15 |  | Hippocampal Pyramidal - Crym | *Crym* | [9] |
| 17 |  | Hippocampal Granule Precursor | *Prox1* & *Nrp2* | [10] |
| 18 |  | Hippocampal Granule | *Prox1* | [10] |
| 2,5,9,26 |  | Cortext Layer5 /layer6 Pyramidal | *Tbr1* & *Pcp4* | [11] |
| 14 |  | Claupyr | *Tbr1* & ^*Pcp4* | [11] |
| 6,13,41 |  | S1PyrDL | *Pcp4* & ^*Tbr1* | [11] |
| 49,52 | Non-neuron | Oligodendroctye | *Pdgfra* | [12] |
| 30,31,39 |  | Oligodendroctye Precursor Cells | *Olig1* & *Olig2* | [13] |
| 47,56 |  | Endothelia | *Flt1* & *Kdr* | [14] |
| 45,58 |  | Smooth Muscle Cells | *Abcc9* & *Pdgfrb* | [15] |
| 32,34,35,21,28,48 |  | Astrocyte | *Gfap* & *Aldh1l1* | [16, 17] |
| 51 |  | Microglia | *C1qb* & *Tgfbr1* | [18] |

**Table S7：**The number of DEGs identified in bulk RNA-seq as the reference for the 10X_5CL and JURKAT_293T datasets.

| Dataset | Cell Lines | DEGs number |
| --- | --- | --- |
| 10X_5CL | A549_H1975 | 4,167 |
|  | H2228_A549 | 4,526 |
|  | H2228_H1975 | 4,090 |
|  | H2228_H838 | 5,629 |
|  | H838_A549 | 4,277 |
|  | H838_H1975 | 4,709 |
|  | HCC827_A549 | 4,310 |
|  | HCC827_H1975 | 3,299 |
|  | HCC827_H2228 | 4,344 |
|  | HCC827_H838 | 4,953 |
| JURKAT_293T | 293T_JURKAT | 5,901 |

## References

1. Deng Y, Bao F, Dai Q, Wu LF, Altschuler SJ: Scalable analysis of cell-type composition from single-cell transcriptomics using deep recurrent learning. *Nat Methods* 2019, 16:311-314.

2. Macosko EZ, Basu A, Satija R, Nemesh J, Shekhar K, Goldman M, Tirosh I, Bialas AR, Kamitaki N, Martersteck EM, et al: Highly Parallel Genome-wide Expression Profiling of Individual Cells Using Nanoliter Droplets. *Cell* 2015, 161:1202-1214.

3. Rosenberg AB, Roco CM, Muscat RA, Kuchina A, Sample P, Yao Z, Graybuck LT, Peeler DJ, Mukherjee S, Chen W: Single-cell profiling of the developing mouse brain and spinal cord with split-pool barcoding. *Science* 2018, 360:176-182.

4. Abraham H, Perez-Garcia CG, Meyer G: p73 and Reelin in Cajal-Retzius cells of the developing human hippocampal formation. *Cereb Cortex* 2004, 14:484-495.

5. Doyle JP, Dougherty JD, Heiman M, Schmidt EF, Stevens TR, Ma G, Bupp S, Shrestha P, Shah RD, Doughty ML, et al: Application of a translational profiling approach for the comparative analysis of CNS cell types. *Cell* 2008, 135:749-762.

6. Alifragis P, Liapi A, Parnavelas JG: Lhx6 regulates the migration of cortical interneurons from the ventral telencephalon but does not specify their GABA phenotype. *J Neurosci* 2004, 24:5643-5648.

7. Thompson CL, Ng L, Menon V, Martinez S, Lee CK, Glattfelder K, Sunkin SM, Henry A, Lau C, Dang C, et al: A high-resolution spatiotemporal atlas of gene expression of the developing mouse brain. *Neuron* 2014, 83:309-323.

8. Xie Z, Ma X, Ji W, Zhou G, Lu Y, Xiang Z, Wang YX, Zhang L, Hu Y, Ding YQ, Zhang WJ: Zbtb20 is essential for the specification of CA1 field identity in the developing hippocampus. *Proc Natl Acad Sci U S A* 2010, 107:6510-6515.

9. Cembrowski MS, Wang L, Sugino K, Shields BC, Spruston N: Hipposeq: a comprehensive RNA-seq database of gene expression in hippocampal principal neurons. *Elife* 2016, 5:e14997.

10. Iwano T, Masuda A, Kiyonari H, Enomoto H, Matsuzaki F: Prox1 postmitotically defines dentate gyrus cells by specifying granule cell identity over CA3 pyramidal cell fate in the hippocampus. *Development* 2012, 139:3051-3062.

11. Zeisel A, Munoz-Manchado AB, Codeluppi S, Lonnerberg P, La Manno G, Jureus A, Marques S, Munguba H, He L, Betsholtz C, et al: Brain structure. Cell types in the mouse cortex and hippocampus revealed by single-cell RNA-seq. *Science* 2015, 347:1138-1142.

12. Marques S, Zeisel A, Codeluppi S, van Bruggen D, Mendanha Falcao A, Xiao L, Li H, Haring M, Hochgerner H, Romanov RA, et al: Oligodendrocyte heterogeneity in the mouse juvenile and adult central nervous system. *Science* 2016, 352:1326-1329.

13. Dai J, Bercury KK, Jin W, Macklin WB: Olig1 Acetylation and Nuclear Export Mediate Oligodendrocyte Development. *J Neurosci* 2015, 35:15875-15893.

14. Tasic B, Menon V, Nguyen TN, Kim TK, Jarsky T, Yao Z, Levi B, Gray LT, Sorensen SA, Dolbeare T, et al: Adult mouse cortical cell taxonomy revealed by single cell transcriptomics. *Nat Neurosci* 2016, 19:335-346.

15. He L, Vanlandewijck M, Raschperger E, Andaloussi Mae M, Jung B, Lebouvier T, Ando K, Hofmann J, Keller A, Betsholtz C: Analysis of the brain mural cell transcriptome. *Sci Rep* 2016, 6:35108.

16. Murthy M, Bocking S, Verginelli F, Stifani S: Transcription factor Runx1 inhibits proliferation and promotes developmental maturation in a selected population of inner olfactory nerve layer olfactory ensheathing cells. *Gene* 2014, 540:191-200.

17. Afsari ZH, Renno WM, Abd-El-Basset E: Alteration of glial fibrillary acidic proteins immunoreactivity in astrocytes of the spinal cord diabetic rats. *Anat Rec (Hoboken)* 2008, 291:390-399.

18. Butovsky O, Jedrychowski MP, Moore CS, Cialic R, Lanser AJ, Gabriely G, Koeglsperger T, Dake B, Wu PM, Doykan CE, et al: Identification of a unique TGF-beta-dependent molecular and functional signature in microglia. *Nat Neurosci* 2014, 17:131-143.
