# Supplementary material for: DISC: a highly scalable and accurate inference of gene expression and structure for single-cell transcriptomes using semi-supervised deep learning
Source: Genome Biol. 2020 Jul 10;21:170. doi: 10.1186/s13059-020-02083-3 (PMC7353747; doi:10.1186/s13059-020-02083-3)
Supplement: Supplementary file 2 — Additional file 2. Review history. [file 13059_2020_2083_MOESM2_ESM.docx]

Review History

**First round of review**

**Reviewer 1**

**Were you able to assess all statistics in the manuscript, including the appropriateness of statistical tests used?**

No

**Were you able to directly test the methods?**

Yes

**Comments to author:**

In this paper, the authors proposed an imputation approach DISC aimed to infer gene structure and expression obscured by dropouts by learning information from both positive and zero-count genes. The authors showed that DISC with the AE-RNN structure is scalable to ultra-large datasets. The inferred gene expression structures and expressions are validated by two independent FISH experiments and down-sampling experiments. This semi-supervised framework is of interest and the evaluation of imputation performance using FISH data is sound. Overall, it is a reasonable paper with complete discussion on the main findings. Here are more comments:

1. For figure 1(D) and 1(E), we usually use methods like PCA on top high variable genes as the input to clustering methods, DISC should be compared with these common methods to show its ability about feature compressed.

2. For figure 2(A, B), it is very nice that DISC runs very fast with a little memory cost, however, with so many hyperparameters in its loss function, is it easy/fast for user to apply Latin Hypercube Sampling? Does the duration of DISC include your hyperparameter optimization method? Because hyperparameter optimization a necessary and time-consuming problem in almost all neural network algorithms. It will be more valuable to clarify them in more details.

3. For figure 3-4, it's very nice that DISC outperforms other methods in those metrics used. But I think the improvement in biological level is more important (e.g. more high variable genes can be found after imputation).

4. For figure 6, you compared the performance of DISC and scScope, however, scScope achieved almost worst scores in the demonstration in figure 4 and figure 5. Because it is the improvement in biological significance, I think more methods should be compared as long as the memory allows.

5. scScope also integrates an autoencoder and a RNN to impute the scRNA-seq data. In my view, I think the scScope is also belonged to the semi-supervised learning. Although the performance of DISC is consistently better than the scScope, it is better if the authors could discuss the differences between these two approaches in the 'Introduction' section.

6. Fig. 2B is hard to understand. More explanations about the workflow should be added in this picture. And I guessed that the "y" in Fig. 2B (1) is in the wrong position.

7. In line 469, change "α_r" to "α_R".

8. In line 434, "The latent representations over all steps, z_c∈~^(L×N)", what are L and N. I think it should be S×T.

**Reviewer 2**

**Were you able to assess all statistics in the manuscript, including the appropriateness of statistical tests used?**

Yes: This paper is somewhat methods heavy. I did not understand a few details (line 420 about 'one expression distribution' and (line 444) on compressor - which i have asked for further clarification).

**Were you able to directly test the methods?**

No

**Comments to author:**

The authors here present a novel deep learning imputation model, DISC, to handle dropout events in single cell RNA-seq analysis. DISC is an auto-encoder (AE) in a Recurrent Neural Network (RNN) framework model that iteratively learns the expression pseudo-counts in a semi-supervised framework. In the model, the learnt low-dimensional representation in AE is used to update the pseudo-counts of originally non-zero values in an RNN framework - to generate the prediction matrix, which is then again fed into AE, thereby iteratively updating the non-zero labels.

The authors have comparatively assessed the imputation accuracy and performance of DISC by comparing against seven other imputation algorithms, which include four other NN-based models, and have shown that:

(i) when comparing against single cell FISH for two datasets, DISC generally outperforms other methods in recovering gene expression structures;

(ii) when compared against other methods in a down-sampling in silico experiment, DISC recovers the dropout events better;

(iii) when compared against other methods in cell type identification problem in 30% and 50% downsampled data, DISC improved identification of major and rare cell types across data generated from different single cell platforms, more than the other methods (some of which failed due to out-of-memory errors); and

(iv) DISC can handle ultra-large datasets (with 1.3 million cells), while being able to recover cell types and beating the existing algorithms in both computational time and memory usage. This efficiency in time and being able to run under limited memory while handling large scale datasets appears to be unique to DISC.

Overall, DISC appears to be a novel computational tool that can handle some of the existing limitations around imputation. However, please address some of the following questions.

------ Major comments: ------

1) Comparison based on downstream steps:

The authors have discussed that DISC compares better than other methods in identifying cell types post-imputation. However, users of the method will still be curious as to whether they should use the imputed results for downstream analyses as well - such as identifying differentially expressed genes, and doing trajectory analyses - which might be the primary focus of many users. Recent, imputation benchmarking manuscript (https://www.biorxiv.org/content/10.1101/2020.01.29.925974v1) has identifed that better imputation and correlation with bulk expression does not necessarily translate well to higher specificity in DEG identification or 'better' downstream trajectories.

While, clustering and trajectory analyses potentially suffer less from overall dropout since the low-dimensional representation can potentially capture the overall cell-cell relationship, identification of DEG is an important benchmarking step for users to better understand the effectiveness of a new imputation method. For users, it is important to understand how the DEG list overlap for large versus smaller log-fold change differences, for instance. If the authors could also show that DISC can outperform other state-of-the-art methods in DEG analyses and preferably, also downstream clustering and trajectory analyses, that would attract more users. Please add these analyses, as much as feasible.

2) Technical versus biological dropouts:

DISC uses a data reconstruction approach using an RNN deep-learning framework to impute the non-zero counts. DISC appears to be different from algorithms like MAGIC and SAVER in that it does not alter non-zero values, however the way it is designed, DISC is agnostic of biological versus technical dropouts. There are algorithms like scImpute that attempt at treating these separately - although this study has also highlighted its limitations. Regardless, potential users of DISC would likely wonder about this as well: Under the high-level current modeling framework, would it be possible to weigh/focus more on imputing the potentially technical dropouts? Please comment.

3) Comparing with sorted bulk expression:

While the analyses does show that DISC does outperform other methods, downsampling to 30% and 50%, being somewhat simulation studies, might not reflect true empirical baseline across these methods. For instance, methods that try to capture gene-gene correlation more might be disfavored under heavily downsampled dataset. In this regard, if the authors could also show comparative analyses without downsampling the original data, that can favor DISC. For instance, similar to scFISH for a handful of genes - which is indeed impressive for DISC, comparing transcriptome-level imputed profile across the methods against bulk expression for sorted cells could be one measure.

----- Minor comments: ------

1. Regarding model architecture and description:

(i) (Figure 1B) Please annotate the matrices in Figure 1B - for example with 'Imputer output' (for the heatmap in 1)/ 'predictor output' (for heatmap in 3); it will help better clarify the label learning process. (Also, is there a meaning to the "white arrow"? And should there be an arrow going from matrix in 3 back to the matrix in 1?)

(ii) (line 613-617) The equation for MAE (mean absolute error) is missing in line 613. Please update the other lines as well.

Other minor details:

- (line 389) In the outlier detection, z-scores greater than 3 are treated as zero-count genes during training, where z-transformation is done across cells for any gene. Not clear why z > 3 would translate to zero-count gene. Please explain/correct.

- (line 420-421) What does 'one expression distribution' mean?

- (line 437-445) In the discussion on compressor autoencoder:

(i) (line 438) z_c -> z_c (with tilde) to match the labels in Figure 1C.

(ii) (line 441 to 445) Is the cell expression profile y_c (given by 443) reversed from z_c (from line 438)? (If yes, please also convert this to z_c with tilde.) This wasn't clear.

2. For the cell type identification using marker genes, how did the authors handle any conflict between two cell types or a case without any clear marker? (Please mention in the methods - also if there were no such case.)

3. (Minor typos) Please fix the typos and grammatrical errors. A few are listed below:

- (line 28) "severer" -> more severe;

- (line 157-158) "is less suffered from" -> "suffers less from"

- (line 168) "Compared the .." -> "When the distributions .. are compared, .."

- (line 228) "which" -> "while"

- (line 276) "Followed the" -> "Following the"

- (line 416) Missing subscript 'c'

4. (Other minor) Please add some additional details in the main text:

- Please mention the number of genes (19 for MELANOMA, 33 for SSCORTEX) with data in scFISH in the main text as well; these are only mentioned in the figure caption.

- (line 255 - 284) In the initial discussion of cell type identifications, please mention that this is for when the samples were downsampled to 30%. This was not clear until after the next paragraph.

**Authors Response**

**Point-by-point responses to the reviewers’ comments:**

Reviewer #1：

In this paper, the authors proposed an imputation approach DISC aimed to infer gene structure and expression obscured by dropouts by learning information from both positive and zero-count genes. The authors showed that DISC with the AE-RNN structure is scalable to ultra-large datasets. The inferred gene expression structures and expressions are validated by two independent FISH experiments and down-sampling experiments. This semi-supervised framework is of interest and the evaluation of imputation performance using FISH data is sound. Overall, it is a reasonable paper with complete discussion on the main findings.

*Thanks very much for the insightful comments, which help us improve the study significantly. We have addressed all the comments below and modified the manuscript accordingly.*

1. For figure 1(D) and 1(E), we usually use methods like PCA on top high variable genes as the input to clustering methods, DISC should be compared with these common methods to show its ability about feature compressed.

*Author’s answer:*

*Thanks for the advice. We actually used top 30 PCs by PCA transformation from the top 2000 highly variable genes of the RETINA dataset as the input to clustering methods to produce figure 1(D). We are sorry that we did not correctly state it. We have changed the title of figure 1(D) accordingly:*

*"(D) T-SNE visualization and clustering using top 30 PCs generated by PCA transformation from the selected top 2000 highly variable genes (HVGs) of the RETINA dataset (ACC=0.950)."*

2. For figure 2(A, B), it is very nice that DISC runs very fast with a little memory cost, however, with so many hyperparameters in its loss function, is it easy/fast for user to apply Latin Hypercube Sampling? Does the duration of DISC include your hyperparameter optimization method? Because hyperparameter optimization a necessary and time-consuming problem in almost all neural network algorithms. It will be more valuable to clarify them in more details.

*Author’s answer:*

*Because hyperparameter optimization is not an easy/fast task for users, we provided a set of hyperparameters as the default so that users can easily use DISC without to undergo the time-consuming optimization process. We tested the default hyperparameter set for many high-throughput single cell datasets with different cell numbers (thousands to millions), different platforms, different cell compositions and the performance was robust as demonstrated in the manuscript. We believe that the set of default hyperparameters will be able to handle most of the single cell sequencing datasets. On the other hand, users are still able to change hyperparameters through command line interface. We have added a sentence in the hyperparameter optimization section to explain the options for users:*

*" We provided a set of hyperparameters as the default so that users can easily use DISC without to undergo the time-consuming optimization process. We tested the default hyperparameter set for many high-throughput single cell datasets with different cell numbers (thousands to millions), different platforms, different cell compositions and the performance was robust. …. Users are also able to change them via command line interface."*

3. For figure 3-4, it's very nice that DISC outperforms other methods in those metrics used. But I think the improvement in biological level is more important (e.g. more high variable genes can be found after imputation).

*Author’s answer:*

*Thanks for the advice, gaining more biological insights is very important for imputation methods. A similar suggestion has also been given by the reviewer 2. Hence, we have added a new section "DISC improves downstream analysis" to evaluate whether DISC improves single cell downstream analyses, including differentially expressed genes (DEGs) identification and trajectory inference, to provide more biological meaningful information. DISC achieved the top performance on DEG analysis and also worked well on trajectory inference.*

4. For figure 6, you compared the performance of DISC and scScope, however, scScope achieved almost worst scores in the demonstration in figure 4 and figure 5. Because it is the improvement in biological significance, I think more methods should be compared as long as the memory allows.

*Author’s answer:*

*Thanks for the advice. We evaluated the scalability of the imputation methods in Figure 2 and evaluated the reliability of imputation results for ultra-large datasets in Figure 6. We tested all the deep-learning methods, including scVI, DCA, DeepImpute, scScope and DISC. We were only able to complete the clustering using scScope and DISC.*

5. scScope also integrates an autoencoder and a RNN to impute the scRNA-seq data. In my view, I think the scScope is also belonged to the semi-supervised learning. Although the performance of DISC is consistently better than the scScope, it is better if the authors could discuss the differences between these two approaches in the 'Introduction' section.

*Author’s answer:*

*Semi-supervised is a training strategy where a model not only learns from the labeled data, but also from the unlabeled data. Although DISC and scScope have similar network structure, they are trained by different strategies. scScope is trained in a supervised-manner, because its loss function is computed only on positive-count genes (in its methods, it states "the entry-sampling operator PZc forces loss computation only on non-zero entries of xc"). In contrast, the loss function of DISC is computed on both positive-count genes (real labels) and zero-count genes (pseudo labels). We discussed this in detail in the Discussion section:*

*" Although DISC and scScope have similar network structure, they are trained by different strategies. DISC employs semi-supervised learning and its loss function is computed on both positive-count genes (real labels) and zero-count genes (pseudo labels), while scScope is trained in a supervised-manner and its loss function is computed only on positive-count genes. As a consequence, DISC distinguishes the technical zero generated by down-sampling (Figures S13A and S14A) and scScope was only able to distinguish technical zero generated by down-sampling in RETINA and BRAIN_SPLiT datasets (Figures S13B and S14B), which contain a large number of cells and positive-count genes. Hence, DISC works well when information provided by positive-count genes is limited."*

6. Fig. 2B is hard to understand. More explanations about the workflow should be added in this picture. And I guessed that the "y" in Fig. 2B (1) is in the wrong position.

*Author’s answer:*

*Thanks for the suggestion and pointing out the error. We changed the layout of Figure 1(B) and gave more explanations in the figure to improve the clarity of the figure.*

7. In line 469, change "α_r" to "α_R"; In line 434, "The latent representations over all steps, z_c∈~^(L×N)", what are L and N. I think it should be S×T.

*Author’s answer:*

*Thanks for the suggestions, we changed it accordingly.*

Reviewer #2：

The authors here present a novel deep learning imputation model, DISC, to handle dropout events in single cell RNA-seq analysis. DISC is an auto-encoder (AE) in a Recurrent Neural Network (RNN) framework model that iteratively learns the expression pseudo-counts in a semi-supervised framework. In the model, the learnt low-dimensional representation in AE is used to update the pseudo-counts of originally non-zero values in an RNN framework - to generate the prediction matrix, which is then again fed into AE, thereby iteratively updating the non-zero labels.

The authors have comparatively assessed the imputation accuracy and performance of DISC by comparing against seven other imputation algorithms, which include four other NN-based models, and have shown that:

(i) when comparing against single cell FISH for two datasets, DISC generally outperforms other methods in recovering gene expression structures;

(ii) when compared against other methods in a down-sampling in silico experiment, DISC recovers the dropout events better;

(iii) when compared against other methods in cell type identification problem in 30% and 50% downsampled data, DISC improved identification of major and rare cell types across data generated from different single cell platforms, more than the other methods (some of which failed due to out-of-memory errors); and

(iv) DISC can handle ultra-large datasets (with 1.3 million cells), while being able to recover cell types and beating the existing algorithms in both computational time and memory usage. This efficiency in time and being able to run under limited memory while handling large scale datasets appears to be unique to DISC.

Overall, DISC appears to be a novel computational tool that can handle some of the existing limitations around imputation. However, please address some of the following questions.

*Thanks very much for the insightful comments, which help us improve the study significantly. We have addressed all the comments below and modified the manuscript accordingly.*

1. Comparison based on downstream steps:

The authors have discussed that DISC compares better than other methods in identifying cell types post-imputation. However, users of the method will still be curious as to whether they should use the imputed results for downstream analyses as well - such as identifying differentially expressed genes, and doing trajectory analyses - which might be the primary focus of many users. Recent, imputation benchmarking manuscript has identified that better imputation and correlation with bulk expression does not necessarily translate well to higher specificity in DEG identification or 'better' downstream trajectories.

While, clustering and trajectory analyses potentially suffer less from overall dropout since the low-dimensional representation can potentially capture the overall cell-cell relationship, identification of DEG is an important benchmarking step for users to better understand the effectiveness of a new imputation method. For users, it is important to understand how the DEG list overlap for large versus smaller log-fold change differences, for instance. If the authors could also show that DISC can outperform other state-of-the-art methods in DEG analyses and preferably, also downstream clustering and trajectory analyses, that would attract more users. Please add these analyses, as much as feasible.

*Author’s answer:*

*Thanks very much for the insightful advice. We have added a new section "DISC improves downstream analysis" in the manuscript to include these analyses.*

*For DEG identification, we evaluated: (1) the overlap of DEGs identified from the same two cell types between the bulk data and scRNA-seq data; (2) the number of false detected DEGs using cells from a homogeneous population; and (3) impact of the magnitude of expression difference by fold-change (FC) on an imputation method. The results were shown in Figure 6, Figures S9 and S10. In general, DISC outperformed the other state-of-the-art methods for two datasets tested and consistently improved DEG identification over different log-fold change intervals.*

*For trajectory inference, we used Monocle2 to infer the pseudo-temporal order for cells and compared with the known cell differentiation order. The results were shown in Figure S11. Consistent with the previous benchmarking paper, most imputation methods did not show a significant improvement over the raw dataset. In our evaluation, DCA, DISC, scVI and MAGIC had slightly improvement over the raw dataset.*

2. Technical versus biological dropouts:

DISC uses a data reconstruction approach using an RNN deep-learning framework to impute the non-zero counts. DISC appears to be different from algorithms like MAGIC and SAVER in that it does not alter non-zero values, however the way it is designed, DISC is agnostic of biological versus technical dropouts. There are algorithms like scImpute that attempt at treating these separately - although this study has also highlighted its limitations. Regardless, potential users of DISC would likely wonder about this as well: Under the high-level current modeling framework, would it be possible to weigh/focus more on imputing the potentially technical dropouts? Please comment.

*Author’s answer:*

*Thanks for the suggestions. Followed the analysis conducted in the SAVER paper, we compared the expression distributions between true zero entries (zero-count genes in RAW) and sampled zero entries (positive-count genes in RAW becomes zero-count genes after sampling in down-sampled data). Because true zeros contain both technical and biological zeros while sampled zeros contain technical zeros only, we expected that their distributions will be different after imputation. The results are shown in figure S13 and S14. The two distributions of true and sampled zeros showed a clear difference after imputation by DISC (Figures S13A and S14A), indicating that DISC handles biological and technical dropouts differently. In addition, to address the suggestions given by the reviewer 1, we also compared the recovery of technical zeros of DISC and scScope. We have added a paragraph in the Discussion section to discuss this:*

*“DISC employs semi-supervised learning and its loss function is computed on both positive-count genes (real labels) and zero-count genes (pseudo labels), while scScope is trained in a supervised-manner and its loss function is computed only on positive-count genes. As a consequence, DISC distinguishes the technical zero generated by down-sampling (Figures S13A and S14A) and scScope was only able to distinguish technical zero generated by down-sampling in RETINA and BRAIN_SPLiT datasets (Figures S13B and S14B)....”*

3. Comparing with sorted bulk expression:

While the analyses do show that DISC does outperform other methods, downsampling to 30% and 50%, being somewhat simulation studies, might not reflect true empirical baseline across these methods. For instance, methods that try to capture gene-gene correlation more might be disfavored under heavily downsampled dataset. In this regard, if the authors could also show comparative analyses without downsampling the original data, that can favor DISC. For instance, similar to smFISH for a handful of genes - which is indeed impressive for DISC, comparing transcriptome-level imputed profile across the methods against bulk expression for sorted cells could be one measure.

*Author’s answer:*

*Thanks for the advice. Using the sorted cell line (Jurkat and 293T) datasets, we calculated Spearman correlation coefficient (SCC) between the imputed scRNA-seq profiles and the bulk RNA-seq profiles for a cell line, as well as for the expression difference between the two cell lines. The results are shown in Figure S8. Overall, all the methods preserved the SCC between cell lines and four methods (DISC, scVI, DCA and VIPER) preserved the correlation between the expression difference, while DISC had the highest improvement. We added a paragraph to discuss this result:*

*"Firstly, we calculated Spearman correlation coefﬁcient (SCC) between the imputed scRNA-seq profiles and the bulk RNA-seq profiles for a same cell line and for the expression difference between two cell lines. All the imputation methods preserved the correlation between scRNA-seq profiles and bulk RNA-seq profiles (Figure S8A). But, only four methods preserved the correlation between the expression difference across the two cell lines of scRNA-seq profile and that of bulk RNA-seq profile, while DISC had the greatest improvement, improving 0.584 of RAW to 0.611 (Figure S8B) indicating the ability of DISC to capture the expression difference between cell types.*

4. (Figure 1B) Please annotate the matrices in Figure 1B - for example with 'Imputer output' (for the heatmap in 1)/ 'predictor output' (for heatmap in 3); it will help better clarify the label learning process. (Also, is there a meaning to the "white arrow"? And should there be an arrow going from matrix in 3 back to the matrix in 1?)

*Author’s answer:*

*Thanks for the advice. We have added the annotation to the matrices and the white arrow as well as another white arrow going from predictor outputs to imputer output.*

5. (line 613-617) The equation for MAE (mean absolute error) is missing in line 613. Please update the other lines as well.

*Author’s answer:*

*Thanks for pointing out this error. There was some compatibility issue between Word and wmf-formatted formula pictures. We have fixed the problem.*

6. (line 389) In the outlier detection, z-scores greater than 3 are treated as zero-count genes during training, where z-transformation is done across cells for any gene. Not clear why z > 3 would translate to zero-count gene. Please explain/correct.

*Author’s answer:*

*We are sorry for the confusion. DISC does not impute outliers so that outliers stay unchanged in the imputed expression matrix. During training, DISC masks outliers and uses semi-supervised learning framework to assign pseudo-counts for the outliers for training. We added a sentence to explain this:*

*" Genes with Z scores greater than three are treated as outliers. DISC does not impute outliers so that outliers stay unchanged in the imputed expression matrix. During training, DISC masks outliers and uses semi-supervised learning framework to assign pseudo-counts for the outliers for training."*

7. (line 420-421) What does 'one expression distribution' mean?

*Author’s answer:*

*We are sorry to use the term “one expression distribution” which caused confusion. Here we assumed that a given gene followed the same expression distribution across most of cells and defined this as “major expression distribution”. We have modified the manuscript accordingly.*

*"We assumed that a given gene followed the same expression distribution across most of cells and defined this as “major expression distribution”. Before sigmoid activation, scaled exponential linear unit (24) (SELU) activation is used to make the channel selection biased the first channel, where the first channel represents the major expression distribution."*

8. (line 437-445) In the discussion on compressor autoencoder:

(i) (line 438) z_c -> z_c (with tilde) to match the labels in Figure 1C.

(ii) (line 441 to 445) Is the cell expression profile y_c (given by 443) reversed from z_c (from line 438)? (If yes, please also convert this to z_c with tilde.) This wasn't clear.

*Author’s answer:*

*Thanks for the advice and we have corrected the mistakes.*

9. For the cell type identification using marker genes, how did the authors handle any conflict between two cell types or a case without any clear marker? (Please mention in the methods - also if there were no such case.)

*Author’s answer:*

*Thanks for the comment. We defined clusters with these cases as unknown cell types. We have added a note in Methods:*

*"When the cluster-specific marker genes contain the reference cell type marker genes, we defined the cluster as the reference cell type. However, if multiple reference marker genes for different cell types, or no reference marker genes appeared in the cluster-specific marker genes, we defined these clusters as unknown cell types. "*

10. (Minor typos) Please fix the typos and grammatical errors. A few are listed below:

- (line 28) "severer" -> more severe;

- (line 157-158) "is less suffered from" -> "suffers less from"

- (line 168) "Compared the .." -> "When the distributions .. are compared, .."

- (line 228) "which" -> "while"

- (line 276) "Followed the" -> "Following the"

- (line 416) Missing subscript 'c'

*Author’s answer:*

*Thanks very much for correcting our typo and grammatical errors. We have corrected them accordingly.*

11. (Other minor) Please add some additional details in the main text:

- Please mention the number of genes (19 for MELANOMA, 33 for SSCORTEX) with data in smFISH in the main text as well; these are only mentioned in the figure caption.

*Author’s answer:*

*We have added the information of FISH data to the main text:*

*"Two independent datasets containing both FISH and scRNA-seq measurements were tested, where the MELANOMA and SSCORTEX datasets have 19 and 33 overlapped genes with FISH, respectively (see Methods for the description of the datasets).”*

12. (line 255 - 284) In the initial discussion of cell type identifications, please mention that this is for when the samples were down-sampled to 30%. This was not clear until after the next paragraph.

*Author’s answer:*

*We have added the information to the beginning of cell type identification section.*

**Second round of review**

**Reviewer 1**

The authors have addressed my concerns.

**Reviewer 2**

The authors have addressed most of the major concerns, and I thank the authors for additional follow up analyses on downstream processes that makes the manuscript more attractive. In particular, the authors have highlighted in this revision that: (i) DISC identifies DEGs better compared to many other algorithms - with higher fractional overlap against DEGs identified using multiple bulk RNA-seq, and generally minimized number of false positive DEGs for two different cell lines; and (ii) DISC ranks third amongst methods tested in pseudo-temporal ordering benchmark study (with ~80% of correctly ordered cell pairs). In addition, the authors empirically show DISC inherently identifies technical drop-outs (in down-sampled data) separate from a mix of technical and biological drop-outs (in raw data). This will be useful to know for the users too, although the exact underpinnings of how DISC makes this distinction within the deep-learning framework is not clear (please do discuss this, if it is).

Please address the following additional details in the manuscript.

(Regarding Figure 1B)

Figure 1B has definitely improved. However, I would encourage and suggest the authors make 1B even more clear and consistent with Figure 1A. For instance, please consider the following:

- In Figure 1B, it is clear that imputer output is a weighted average of predictor outputs, as in Figure 1A. Similarly, reconstructor output is a weighted average of the decoder outputs. But, Figure 1B shows a connection between imputer output and decoder outputs, whereas from Figure 1A, decoder outputs are generated from the predictor matrices. So:

- should there be arrows (I suggest three arrows) going from predictor outputs to decoder outputs instead?

- I also suggest breaking this connection between the imputer output and prediction targets in Figure 1B. As I understand it, the prediction targets use decoder outputs and input only. Please see the attached figure showing these edits.

(Regarding pseudotime ordering)

- (line 770-771) Please fix the grammar and rephrase; the latter-half of the statement is not entirely clear.

- (Line 772-776) Please also elaborate on the statement "If a cell has a higher differentiation level in the pair, then the cell must have a higher pseudo-time to define the pair in a correct order", and possibly the next ("Since the root state of the tree defines the pseudo-time for all the cells..(?)") Please provide an example if that is easier.

- Also, for the pseudotemporal ordering, it is also possible that the distance between the 'mis-ordered' pairs could actually less in DISC compared to some other methods, if the authors want to check and mention this.

Other minor comments:

- (Figure S10 caption, line 60) There are no boxplots. Please remove the last line discussing the boxplots.

- (Line 356) Please cite the previous study being mentioned.

**Authors Response**

**Point-by-point responses to the reviewers’ comments:**

Authors’ Response to Reviewers

Reviewer #2：

The authors have addressed most of the major concerns, and I thank the authors for additional follow up analyses on downstream processes that makes the manuscript more attractive. In particular, the authors have highlighted in this revision that: (i) DISC identifies DEGs better compared to many other algorithms - with higher fractional overlap against DEGs identified using multiple bulk RNA-seq, and generally minimized number of false positive DEGs for two different cell lines; and (ii) DISC ranks third amongst methods tested in pseudo-temporal ordering benchmark study (with ~80% of correctly ordered cell pairs). In addition, the authors empirically show DISC inherently identifies technical drop-outs (in down-sampled data) separate from a mix of technical and biological drop-outs (in raw data).

*Thanks very much for the insightful comments, which help us improve the study significantly. We have addressed all the comments below and modified the manuscript accordingly.*

1. This will be useful to know for the users too, although the exact underpinnings of how DISC makes this distinction within the deep-learning framework is not clear (please do discuss this, if it is).

*Author’s answer:*

*Because technical and biological dropouts both belong to zero-count genes, training based only on limited positive-count genes is very likely to miss the distinguishing features between technical and biological dropouts, thus leading to a latent representation that best interprets positive-count genes without properly encodes distinguishing features for dropouts. Semi-supervised learning supplements the training with zero-count genes (dropouts) to build a more reasonable latent representation which also best interprets zero-count genes structurally similarly to positive-count genes as technical dropout. We have added a sentence in the discussion section to explain this:*

*" When positive-count genes are limited, training based only on positive-count genes is likely to miss the distinguishing features between technical and biological dropouts where both belong to zero-count genes, thus leading to a latent representation that best interprets positive-count genes without properly encodes distinguishing features for dropouts. Semi-supervised learning supplements the training with zero-count genes to build a more reasonable latent representation which also best interprets zero-count genes structurally similarly to positive-count genes as technical dropout."*

2. Figure 1B has definitely improved. However, I would encourage and suggest the authors make 1B even more clear and consistent with Figure 1A. For instance, please consider the following:

- In Figure 1B, it is clear that imputer output is a weighted average of predictor outputs, as in Figure 1A. Similarly, reconstructor output is a weighted average of the decoder outputs. But, Figure 1B shows a connection between imputer output and decoder outputs, whereas from Figure 1A, decoder outputs are generated from the predictor matrices. So:

- should there be arrows (I suggest three arrows) going from predictor outputs to decoder outputs instead?

- I also suggest breaking this connection between the imputer output and prediction targets in Figure 1B. As I understand it, the prediction targets use decoder outputs and input only. Please see the attached figure showing these edits.

*Author’s answer:*

*Thanks for the suggestions and we have modified the figure accordingly:*

3. - (line 770-771) Please fix the grammar and rephrase; the latter-half of the statement is not entirely clear.

- (Line 772-776) Please also elaborate on the statement "If a cell has a higher differentiation level in the pair, then the cell must have a higher pseudo-time to define the pair in a correct order", and possibly the next ("Since the root state of the tree defines the pseudo-time for all the cells..(?)") Please provide an example if that is easier.

- Also, for the pseudotemporal ordering, it is also possible that the distance between the 'mis-ordered' pairs could actually less in DISC compared to some other methods, if the authors want to check and mention this.

*Author’s answer:*

*Thanks very much for the suggestions. We have rewritten the trajectory analysis section to make the procedure clearer:*

*" Briefly, each cell in BONE_MARROW was marked as one of the 13 hematopoietic cell types whose bulk RNA-seq profile has the highest Spearman correlation with the cell’s scRNA-seq profile. "*

*and*

*"For example, for a pair of cells, a HSC and a Monocyte, the order of the pair is correctly inferred if the Monocyte is marked a higher pseudo-time than the HSC since the Monocyte has a higher differentiation level than the HSC. Since the root state can be any of the leaves of the constructed trajectories tree and the root state determines the pseudo-time for all the cells (root state is the pseudo-time 0). Hence, the overall percentage of correctly inferred cell pairs for all the possible cell pairs was calculated for all possible root state and the highest percentage was used for comparison. "*

*We also check the distance between the 'mis-ordered' pairs and DISC significantly reduced distance. We add a sentence to mention this result in the downstream analysis section:*

*"In addition, the average distance between the mis-ordered cell-pairs was reduced by DISC to 1.65 from 2.31 of RAW (DISC was ranked the second and close to the best score of DCA (1.61). "*

4. - (Figure S10 caption, line 60) There are no boxplots. Please remove the last line discussing the boxplots.

- (Line 356) Please cite the previous study being mentioned.

*Author’s answer:*

*Thanks for pointing out the mistakes, we have changed them accordingly.*
